# Critical analysis of *Echinacea* preparations marketed in Germany

**DOI:** 10.1007/s00210-024-03634-2

**Published:** 2024-11-28

**Authors:** Carina Groß, Roland Seifert

**Affiliations:** Institute of Pharmacology, Hannover Medical School, Carl-Neuberg-Str. 1, 30625 Hannover, Germany

**Keywords:** *Echinacea*, *Echinacea* pressed juice, *Echinacea* extract, Standardization, Common cold, *E. purpurea*, *E. pallida*, Alkamides, Chicory acid, Polysaccharides

## Abstract

Colds are the most common cause of incapacity to work in 2023 and therefore have a significant impact on the German economy. *Echinacea* preparations are considered a non-specific immune stimulant that is used as a phytotherapeutic agent for the treatment and prevention of colds. In the 2021 ZEIT ranking of the best-selling pharmacy-only medicines in Germany, four *Echinacea* preparations were among the top ten, and they experienced a new hype during the *SARS-CoV-2* pandemic. In this study, 22 *Echinacea* monopreparations, which are approved as herbal medicinal products, were analyzed based on their package inserts. The preparations used in clinical studies were compared with the preparations available on the market with regard to the criteria of plant species used, plant part used, preparation (extract or pressed juice), drug-extract ratio, single dose administered, standardization of the preparations, prior performance of an ingredient analysis, galenics, dosage, duration of administration, and daily dose administered. The analysis revealed that the preparations available on the market are very heterogeneous. The composition can vary considerably in terms of ingredients. For customers, these products appear homogeneous in terms of their presentation on the market. The legal framework conditions promote this situation. The clinical studies are also unable to adequately reflect the market situation. New studies based on market reality, with an analysis of ingredients in the preparations used, a standardized study design, and the inclusion of hematological and biochemical parameters in the evaluation should improve this situation. Days of incapacity for work as an outcome parameter could be well suited.

**Supplementary Information:**

The online version contains supplementary material available at 10.1007/s00210-024-03634-2.

## Introduction

In 2023, the number of sick days at work in Germany reached a new high. On average, 55 out of 1000 employees were unable to work every day according to a report in ZEIT Online. The head of DAK, Andreas Storm, classifies this as alarming for the economy, with the work processes of many companies and authorities being impaired. The main problem here is respiratory illnesses — most days of incapacity to work are due to colds (Voigt 2024).

“Home remedies” are advertised for treatment on the Internet. For example, the “Deutsches Ärzteblatt” headlines that *Echinacea* strengthens the immune system and is recommended as a prophylactic against colds (Rösch 2008). The NDR also recommends *E. purpurea* for colds with coughs and sniffles (NDR 2023). *Echinacea* preparations are regarded as a non-specific immunostimulant that is used as a phytotherapeutic agent for the treatment and prevention of colds.

In the last decade, however, popular science journals such as Ökotest (Öko-Test Jahrbuch Gesundheit für 2010) have increasingly expressed doubts about its effectiveness as a result of which products and advertising have disappeared from the market. Nevertheless, four *Echinacea* preparations are among the top ten in the 2021 ZEIT ranking of the best-selling over-the-counter medicines in Germany (Statista 2021). In 2020, a study was published on the *Echinacea* preparation “Echinaforce” from the manufacturer A. Vogel, which showed virucidal activity against coronaviruses in vitro and thus postulated “Echinaforce” as a prophylactic treatment for *SARS-CoV-2* as well (Signer et al. 2020a and b). As a result, this preparation quickly became popular during the *SARS-CoV-2* pandemic and was sold at inflated prices on the Internet. This increased the interest in *Echinacea* preparations again, but it was also criticized that the virucidal effect on coronaviruses was only found in vitro (Budinger 2020). Later studies showed that “Echinaforce” was able to significantly reduce the viral load and also alleviate other cold symptoms (Wegner 2022), even though Kolev et al. (2022) pointed out some limitations in their clinical study.

The effect of *Echinacea* preparations has been and continues to be the subject of many studies, whereby the efficacy has been evaluated varyingly and discussed critically. In 2014, the HMPC (European Medicines Agency 2023a) issued a recommendation for pressed juice from the fresh herb of the *E. purpurea* plant for the short-term prophylaxis and treatment of acute upper respiratory tract infections in adolescents and adults. In the Cochrane review (Karsch-Völk et al. 2014), the study situation is critically assessed; for example, there would be shortcomings in the documentation of the duration of the illness and the relief of the illness. In addition, 70% of the studies used very different preparations that differed in terms of plant species, plant part, or preparation. The Cochrane review therefore concludes that there are no significant results for the prevention and treatment of colds.

This contradictory situation is also characterized by the question of whether *Echinacea* preparations can be regarded as a uniform, homogeneous product at all. The starting material can be the entire above-ground plant, the root, or combinations thereof. These contain different ingredients. For *Echinacea* species or parts thereof, only the dried drug, i.e., the plant material used, is defined in the European Pharmacopoeia. If the dried drug as a raw material meets the specifications of the pharmacopeia, the manufacturer can use different processing methods for his respective preparation. The products can be very different in terms of ingredients and are therefore heterogeneous.

In studies very different preparations were used, making it almost impossible to compare the results. In this paper, the heterogeneity of the *Echinacea* monopreparations available on the market is analyzed on the basis of the package leaflets. This is the basic information available to customers for information about the respective preparation. The preparations used in the clinical studies are also analyzed in order to compare the extent to which the preparations tested in the studies correspond to the market reality. In this context, the results of in vivo and in vitro studies are also included in the work.

## Material and methods

**Fig. 1:**
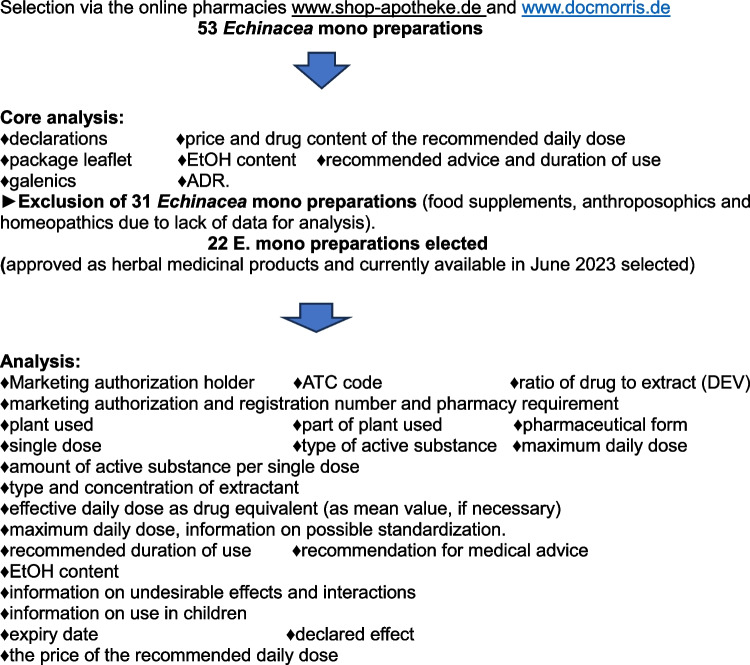
Selection of *Echinacea* preparations to analyze the current market situation in Germany

The selection of *Echinacea* preparations to analyze the current market situation in Germany is summarized in Fig. 1.

**Fig. 2:**
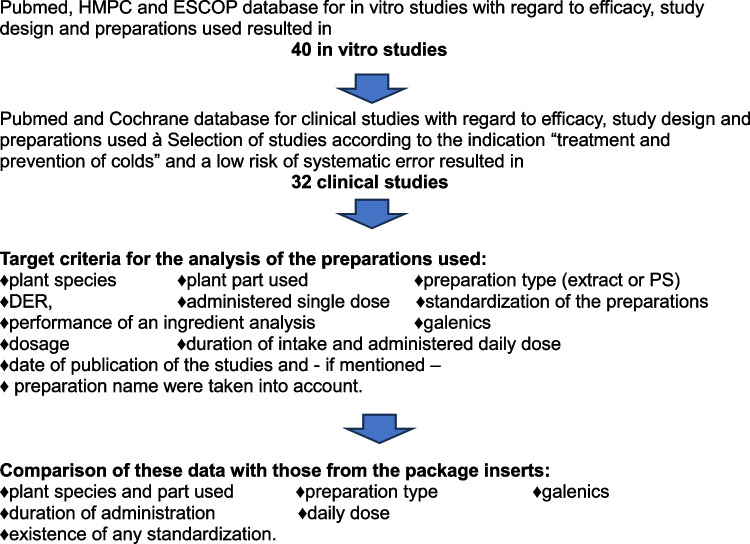
Selection and analysis for the evaluation of the market situation

To evaluate this market situation, studies were selected and analyzed according to the following scheme (Fig. 2).

Thirty-two clinical studies were selected, and they are also included in the Cochrane review or the monographs of HMPC (European Medicines Agency (2023a, b, c) and ESCOP (ESCOP Monographs Online-series 2021). This also reflects the market situation as the monographs/reviews mentioned have a major influence on the marketing authorization of phytotherapeutics, especially the HMPC monograph. While the HMPC monograph included three studies in the positive assessment of PS from *E. echinacea* herba that corresponded exactly to the specified intended use, the Cochrane review included all studies with a low risk of systematic error. The data collected from the package leaflets and in vitro, in vivo, and clinical studies were documented in tabular form as part of the data collection.

Finally, the causes of the differences in the preparations were investigated, starting with the raw plant, the preparation, the extraction agent, and the manufacturing process. All eleven manufacturers were contacted for this purpose.

## Results and discussion

### “Well-established” and “traditional use”

**Fig. 3:**
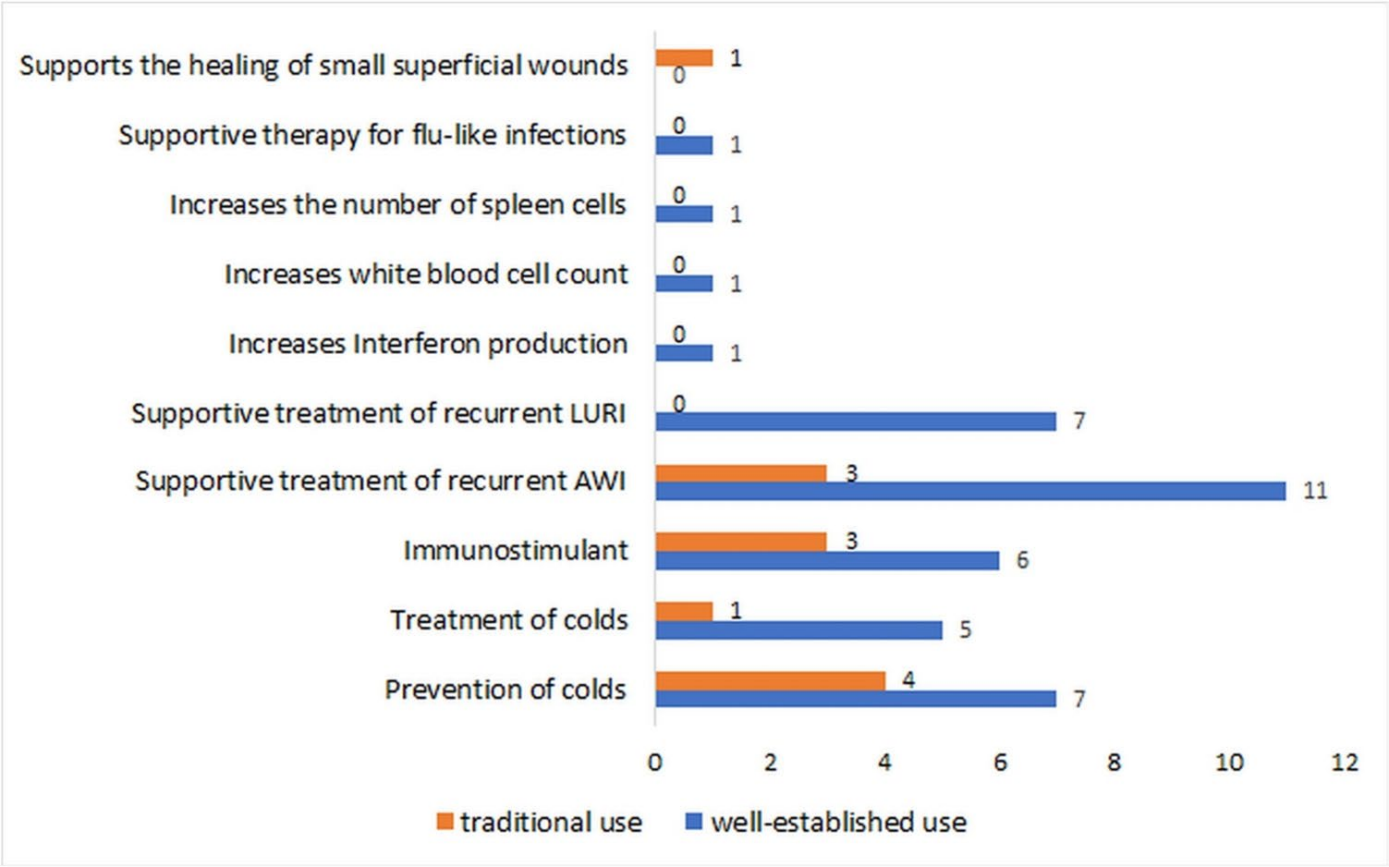
Absolute number of declared effects of *Echinacea* monopreparations by type of authorization/registration

Each of the 22 *Echinacea* preparations analyzed here is approved as a medicinal product. This often gives the impression to customers that the preparations are therefore up-to-date and frequently clinically tested and thus homogenous with regard to the ingredients (Trabert and Seifert 2023). Sixteen of the 22 *Echinacea* products are authorized for well-established use, 6 registered for traditional use. The main named purposes for the preparations are “treatment and prevention of colds” and “immune stimulation.” With regard to the intended use, there were multiple responses in the various preparations (Fig. 3).

The “well-established use market authorization” is based on Article 10a of Directive 2001/83/EC (health.ec.europa.eu 2001a), the “traditional-use market authorization” on Article 16a (1) of Directive 2001/83/EC (health.ec.europa.eu 2001b; Figure S1 Supplement). While in “well-established use” a clinical study (at least bibliographically) must be documented in addition to 10 years of use, at least 30 years of use without evidence of a clinical study is sufficient for “traditional use” (Steinhoff 2008). The development of monographs by the HMPC committee is intended to harmonize the regulations on herbal medicinal products within the EU.

An HMPC monograph with a positive assessment for “well-established use” has only been available for PS from *E. purpurea herba* since 2005. Most *Echinacea* preparations on the market were first identified as medicinal products before the HMPC monographs were established and later confirmed in the post-authorization process depending on the manufacturer’s interest. However, the grandfathering of previously approved, non-compliant products guarantees that they remain on the market (Merz et al. 2016). These are only pharmacopeia-compliant. Since only the raw drug is defined there via a minimum content of lead substances (declaration of the plant species), the composition of ingredients in the end product could be different (European Pharmacopoeia 2020). In phytotherapy, the entirety of the plant’s ingredients represents the active principle (Merz et al. 2016).

### Postulated active ingredients

Accordingly, the “drug” is not defined by specific ingredients, but only by the whole plant part used. Depending on the plant species and parts used and their different processing methods, there are changes in the composition of the ingredients in the respective product.

Polysaccharides, caffeic acid derivatives, alkamides, and ketoalkenes/by-products are considered marker substances to which a pharmacological effect is attributed in the literature. The composition and effect of the preparations also depend on habitat, cultivation, time of harvest, storage, and preparation (Bauer 1998; Bergeron and Gafner 2007; Perry et al. 1997; Perry et al. 2001). Plants grown in China or the USA, for example, differed significantly in their content of alkamides and chicory acid (Luo et al. 2003). The content of ingredients varies seasonally (Bauer 1999). The best time to harvest the herb is the biennial flowering plants.

The proportion of marker compounds also differs depending on the plant species and part. Polysaccharides are found in *E. purpurea* herba, *E. purpurea* radix, and *E. pallida* radix in a similar proportion (Classen et al. 2019). This also applies to caffeic acid derivatives. For *E. purpurea* herba et radix, chicory acid is characteristic. Other caffeic acid derivatives are not necessarily present here (Gilroy et al. 2003). A major difference is that echinacosides are detectable in *E. pallida* radix, which are absent in *E. purpurea*. Other caffeic acid derivatives, on the other hand, do not necessarily occur in *E. pallida* radix (Gilroy et al. 2003). Alkamides are also found in all plants. *E. purpurea*, in particular the root, has a higher content than *E. pallida.* Ketoalken/ines are primarily found in *E. pallida radix* (Osowski et al. 2000).

The type and quantity of ingredients also depend on how the plant material is processed. Caffeic acid derivatives, alkamides, and ketoalkenes/ynes can undergo changes during storage and processing. Caffeic acid derivatives are degraded by the plant’s own phenol oxidases from the time the plants are crushed. To prevent this, the plant material must be filtered or pasteurized at an early stage; the addition of 20% EtOH only slows down the degradation (Nüsslein et al. 2000). Alkamides are rapidly degraded in dried extracts, whereas they are stable in alcoholic solutions (Liu and Murphy 2007). Ketoalken/ines are rapidly oxidized to 8-hydroxy compounds. It is unknown whether the degradation products could have pharmacological significance.

Polysaccharides (e.g., arabinogalactans and fructans of the inulin type) are found in high concentrations in PS without added EtOH (Claasen, 2018). At higher EtOH contents (data vary between 60–65%), they are eliminated. This means that they are only found to a limited extent in PS with a correspondingly high EtOH content and in corresponding extracts (Classen et al. 2019). Caffeic acid derivatives are included in PS and alcoholic extracts. Without stabilization during processing, they can only be found in small quantities in the preparations (Classen et al. 2019). Alkamides are mainly found in extracts with a higher EtOH content due to their lipophilic character. They were therefore only detected in traces in PS without any additives (Classen et al. 2019).

Regardless of their actual effectiveness, the marker substances can serve as an indication of the type of ingredients depending on the type of preparation with regard to the hydrophilic, hydrophobic, and polymeric properties.

### Market analysis: analysis of package inserts

Twenty-two *Echinacea* monopreparations are registered as herbal medicinal products in well-established (16 preparations) or traditional use (6 preparations) and are available on the German market. These were analyzed on the basis of their package leaflets, because this is also the most important information for consumers.

### Plant species and parts used in the preparations

**Fig. 4:**
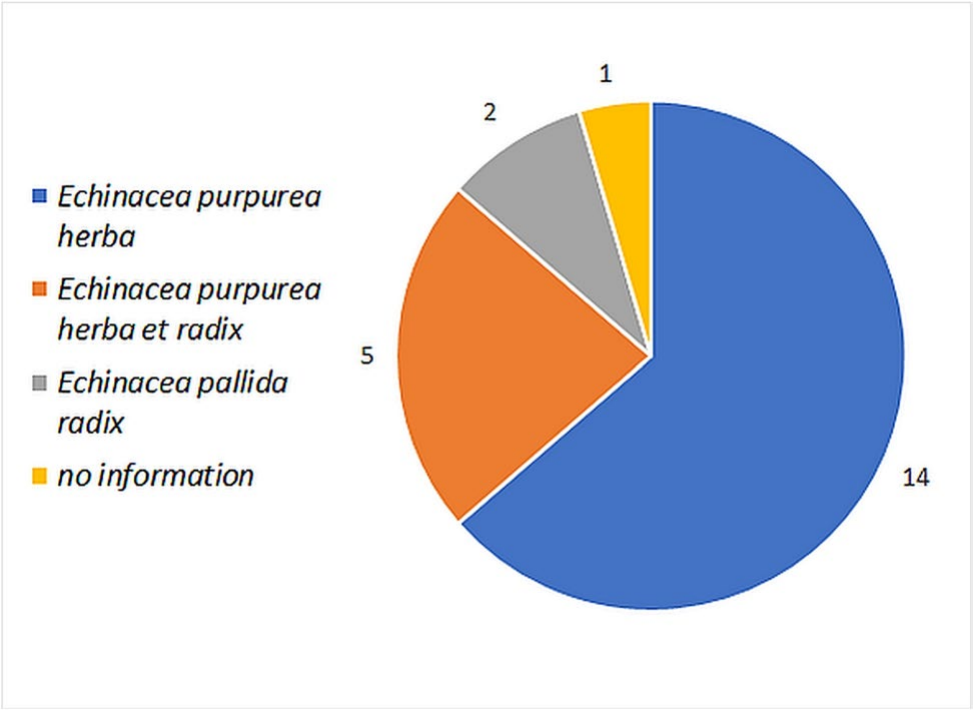
Plant species and parts used in the preparations on the market as absolute numbers

Different plant species and parts are used in the preparations and moreover in different proportions (Fig. 4). Most of the preparations contain only the herb of *E. purpurea*. Preparations containing both the herb and the root of *E. purpurea* in different proportions have the second largest share. Then follow preparations with the root of *E. pallida*.

#### Method of preparation

**Fig. 5:**
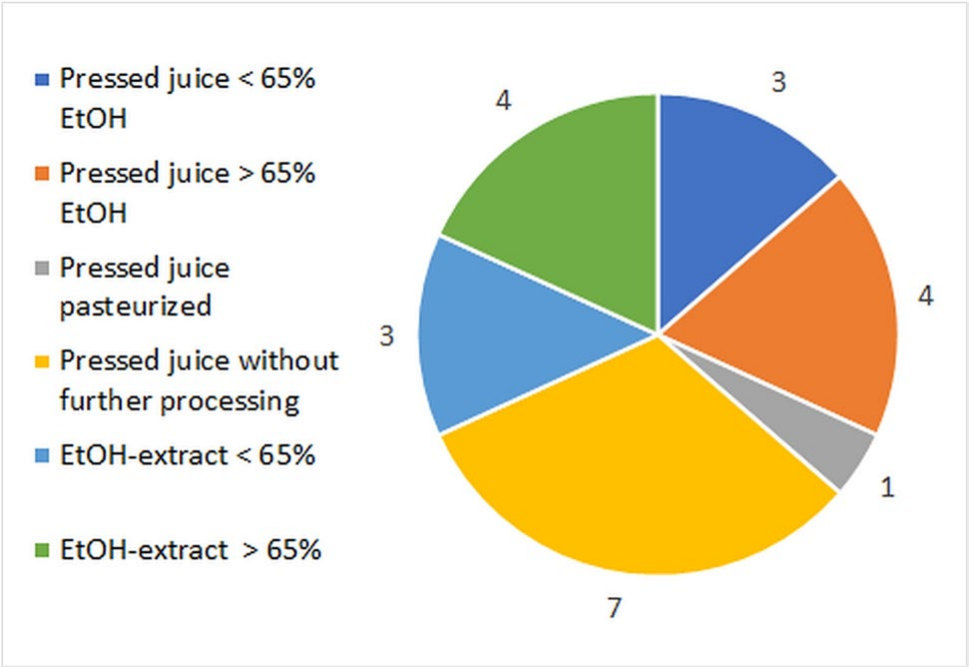
Additives that are added to the preparations on the market during manufacture as absolute numbers

The preparation types of the plant material diverge greatly. In each case, 15 of the preparations contain fresh or dried PS, while extracts account for 7. The analysis of the additives in the products confirms this divergence. Seven of the PS are not subject to any further processing. EtOH is added to 7 of the PS, and 4 contain over 65% EtOH. One product contains pasteurized PS. Of the alcoholic extracts, 4 contain more than 65% EtOH (Fig. 5).

The type and quantity of ingredients differ in connection with the various types of preparation. A direct assessment of the products cannot be made at this point, as scientifically sound statements could only be made experimentally. Ingredient analyses for the marker substances were found for 5 of the preparations of *E. purpurea* mentioned. These included two PS with > 65% EtOH, one PS with < 65% EtOH, one pasteurized PS, and one extract with < 65% EtOH. Both PS with an EtOH content > 65% and the PS with an EtOH content < 65% did not contain any chicory acid. Chicory acid was detected in an EtOH extract with < 65% EtOH as well as in a particularly high concentration of 4000 µg/ml in a PS pasteurized without EtOH. Alkamides were present in a particularly high concentration (12.9 µg/ml) in an EtOH extract < 65% EtOH. Within a range of 0.81–1.2 µg/mL, they were detected in both PS with > 65% EtOH as well as in the PS that had only been pasteurized. The PS with < 65% EtOH had hardly any alkamides. Data on polysaccharides were not given (Osowski et al. 2000).

Even if the herbal drug is defined as a whole, it can therefore be completely heterogeneous in terms of ingredients. Consumer transparency is limited here. For example, studies on the efficacy of alkamides cannot be applied to all products.

### Dose and price

The DER is different in both pressed juices and extracts, and other additives are added to the raw product. Due to the divergent preparation methods, a comparison of the dosage in grams alone is not meaningful and misleading for consumers, pharmacists, and doctors. An approximation of comparability here is the calculation of the effective daily dose as a drug equivalent in grams, and this represents an average value of the DER stated (calculation according to Dingermann 2000).

**Fig. 6:**
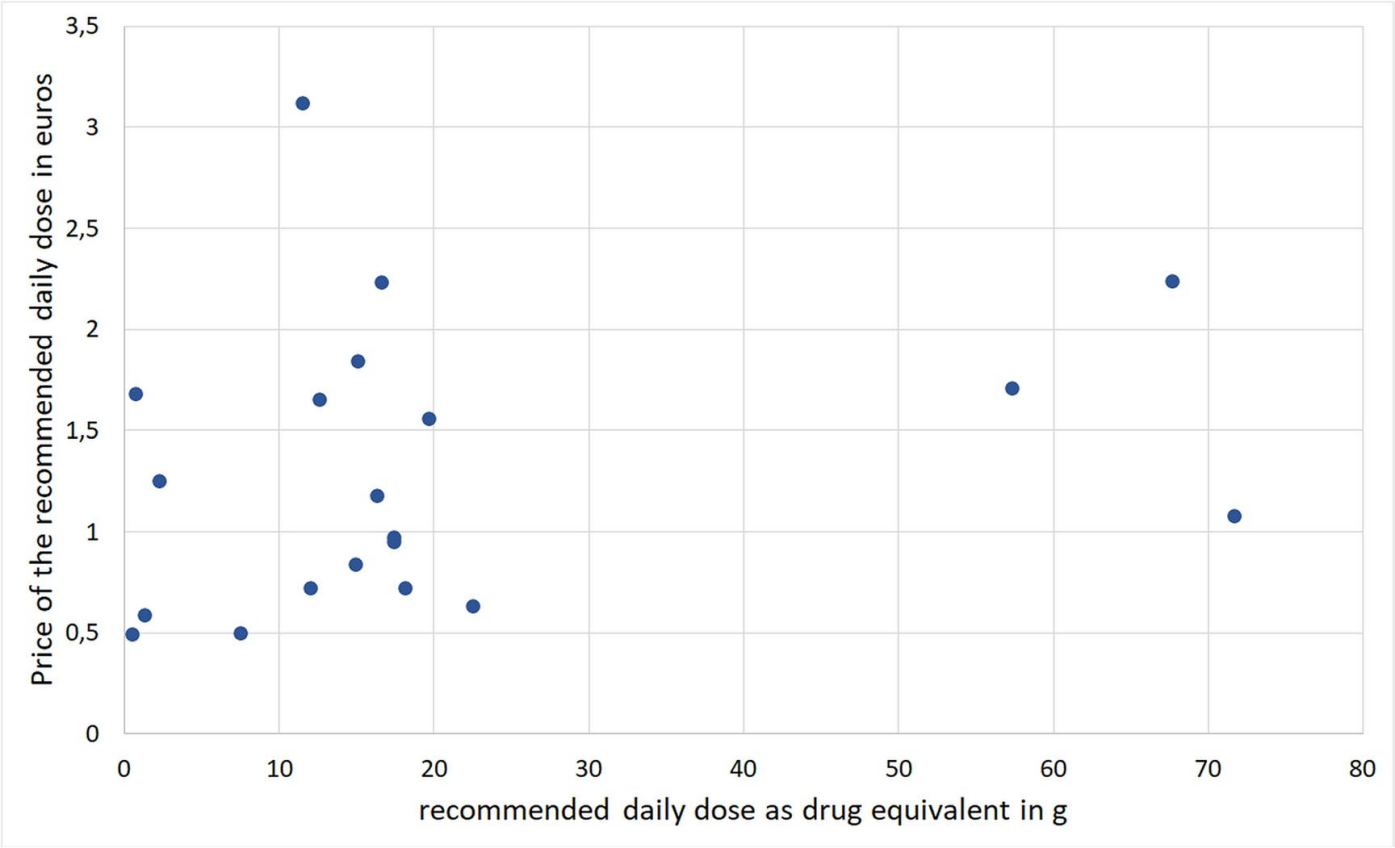
Comparison of the price of the recommended daily dose and the recommended daily dose as a drug equivalent

There is also a wide spread here concerning the products (Fig. 6). There is no recognizable trend that a certain dosage form is associated with a certain amount of drug equivalent. Otherwise, there is a spread from 0.54 g in the lowest-dose product to 71.7 g in the highest-dose product. Eighteen percent of the preparations had a drug content of < 5 g. Twenty-seven percent of the preparations had an average drug content of 5–15 g, 41% of the preparations had a drug content of 15–25 g. Only 14% of the preparations had a drug content of over 25 g.

A comparison of the price/recommended daily dose and the drug content/recommended daily dose shows no clear correlation. The therapeutic range of *Echinacea* preparations would be particularly relevant at this point. This would be a critical point with regard to ADRs with unknown dose information. However, no serious ADRs have been reported to date in the HMPC-Monographs, so that the use within the existing range appears to be safe.

Further information on the analysis of the package leaflets with regard to the number of manufacturers, galenics, recommended duration of use, declared ADR, and interactions as well as the recommendation for use in children can be found in the supplement (Figure S2 – S7).

### In vitro and in vivo studies

The in vitro studies investigated in particular antiviral effects and immunostimulatory or immunomodulatory activity (Figure S8 Supplement). Antiviral effects were detectable for enveloped viruses after pre-incubation of the viruses (Hudson et al. 2005; Pleschka et al. 2009). The results for immune stimulation and modulation were contradictory. On the one hand, the non-specific immune response was stimulated (Burger et al. 1997; Randolph et al. 2003); on the other hand, the immune response in *RV*-infected cells was downregulated (Sharma et al. 2006). Depending on the plant part and extraction method, this effect could also be the opposite (Benson et al. 2010). As another result, despite the observed effects, the effects were rather independent of the marker substances (Vimalanathan et al. 2009); only for alkamides could a concentration dependence be shown in some cases (Hudson et al. 2005). The effective concentrations in the tests were in most cases in the µg–mg range (data not shown).

In a feeding trial on pigs, the immune system was equally activated by the administration of two different *Echinacea* preparations in connection with a vaccination. However, no connection to the marker substances could be established because the commercial preparation used contained no caffeic acid derivatives and only a few alkamides (Maas et al., 2005). In vivo, effects could also be shown for the use of polysaccharides isolated from *Echinacea*, which were, however, injected into the animals in order to make them bioavailable (Roessler et al. 1991).

More detailed data on the in vitro studies can be found in the supplement with information on the plant parts used as well as the preparations used (Figures S8 – S10). Overall, effects could often not be assigned to the marker substances, so that other ingredients and/or synergies appear to be important in addition to the marker substances. Due to the concentrations used in the studies, possible bioavailability after oral ingestion must also be taken into account.

### Bioavailability

With regard to bioavailability, reference can only be made to the marker substances. Until now, bioavailability after ingestion per os has only been clearly demonstrated for alkamides.

Alkamides diffuse passively in the model system with Caco-2 monolayers with an apparent permeability of 3 × 10^−4^ to 3 × 10^−6^ cm/s (complete absorption of a substance is possible at > 1 × 10^−6^ cm/s), while caffeic acid derivatives can only pass through a membrane to a small extent (Jager et al. 2002; Matthias et al. 2005). After oral administration of a commercial preparation (alkamides 9 mg or 0.07 mg), alkamides are already found in the range of 136 or 0.4 ng/ml in human serum after approx. 30 min (Matthias et al. 2007; Woelkart et al., 2006).

Even though the absorption of caffeic acid derivatives depends on the general conditions, (Woelkart and Bauer 2007; Wang et al. 2022; Simonetti et al. 2001), only very low concentrations in human serum should actually be possible, as caffeic acid derivatives are subject to high metabolization (Graefe and Veit 1999) and excretion in the urine (Uang and Hsu 1997).

The bioavailability of polysaccharides is not clear. They are usually degraded in the small intestine to inactive sugar components or are not absorbable due to the size of the molecule. One possibility of interaction may be an interaction with Peyer’s patch cells. Positive effects could be measured in vitro (Bodinet et al. 2004). After feeding mice with high molecular weight melanin from *Echinacea*, there could be found an increase in the production of interleukin-6 and immunglobulin A in these cells (Pugh et al. 2005).

The effect directly in the pharynx on the epithelia is another possibility of direct bioavailability, which has been postulated both in vitro (Signer et al. 2020a; Vimalanathan et al. 2022) and in two clinical studies (Nicolussi et al. 2022).

### Clinical studies — analysis and comparison with the market situation via the package inserts

An analysis of the preparations used on the market (via package inserts) and in clinical studies was carried out to determine whether the preparations used in the clinical studies reflect the market situation and whether conclusions can be drawn about efficacy.

**Fig. 7:**
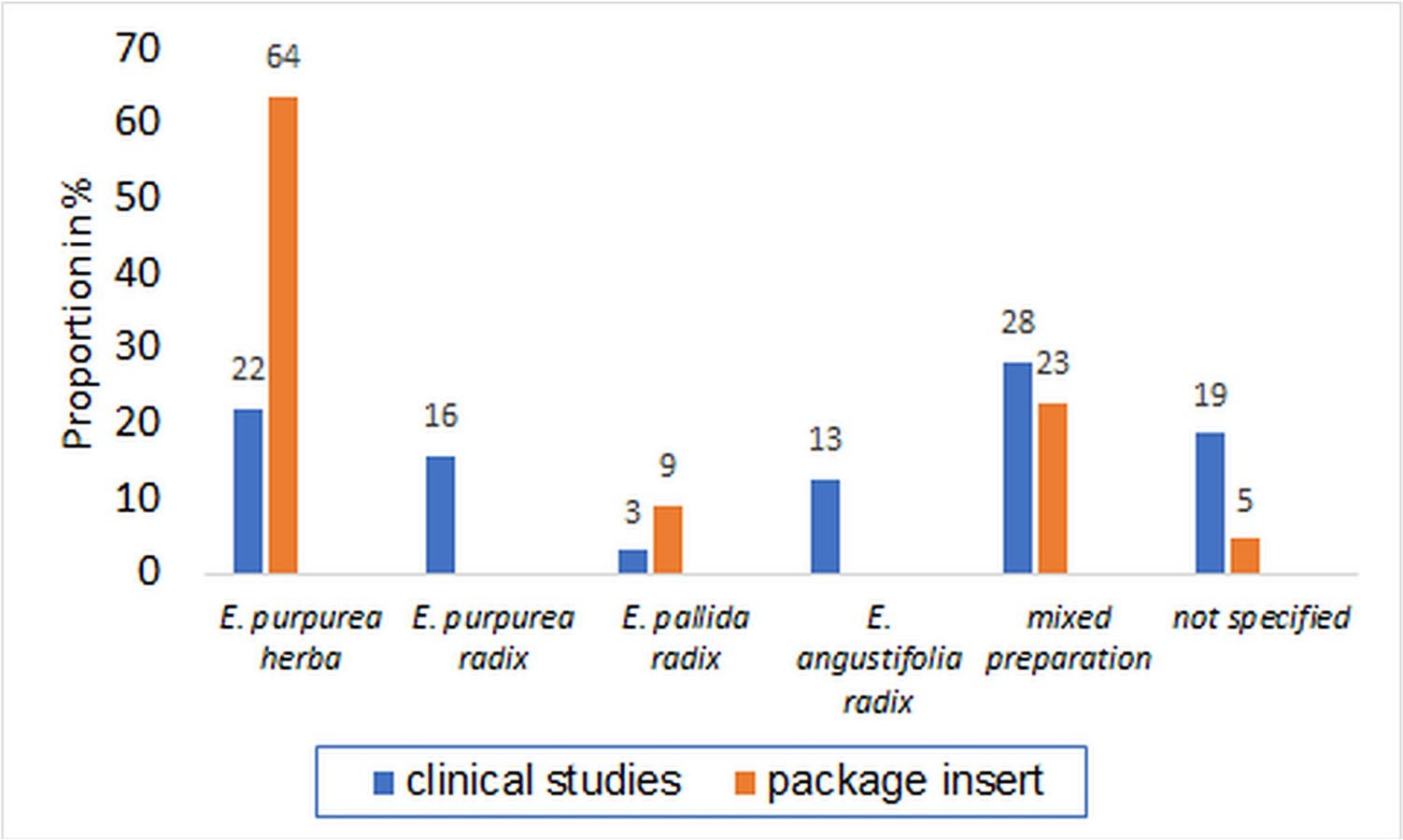
Comparison of the plant parts used in the preparations in clinical studies with the preparations available on the market

In 19% of the studies, there is no precise information on the plant parts used (Fig. 7). This is only the case in 5% of the market products. Compared to the products on the market, preparations of *E. pallida* radix are used in only a few studies. *E. purpurea* radix and *E. angustifolia* radix are not available on the market as monopreparations. Mixed preparations are found in similar proportions in clinical studies and on the market (*E. purpurea* herba et radix). Preparations with *E. purpurea* herba dominate the market. In clinical studies, however, only 22% are based on *E. purpurea* herba.

A large proportion of the studies does not specify which parts of the plant were used. This lack of transparency makes an assessment impossible, as no basic statement is made about possible ingredients. The use of a monopreparation of *E. purpurea* radix appears positive in order to enable statements to be made about the proportion of effect in mixed preparations of *E. purpurea* herba et radix. In addition, *E. pallida* has different ingredient spectra due to the echinacoside content, which is not found in *E. purpurea*, and little can be transferred from the study results to products with *E. purpurea*. Focusing on *E. purpurea* preparations in clinical studies, which are represented with the main share of the market, could improve the validity.

In clinical studies, mixed preparations from several plant species are sometimes used. Such preparations are not available on the market. The respective contribution to the overall effect has not been proven in these cases. As there is no clear evidence of an effect, the benefit of these studies must remain open.

**Fig. 8:**
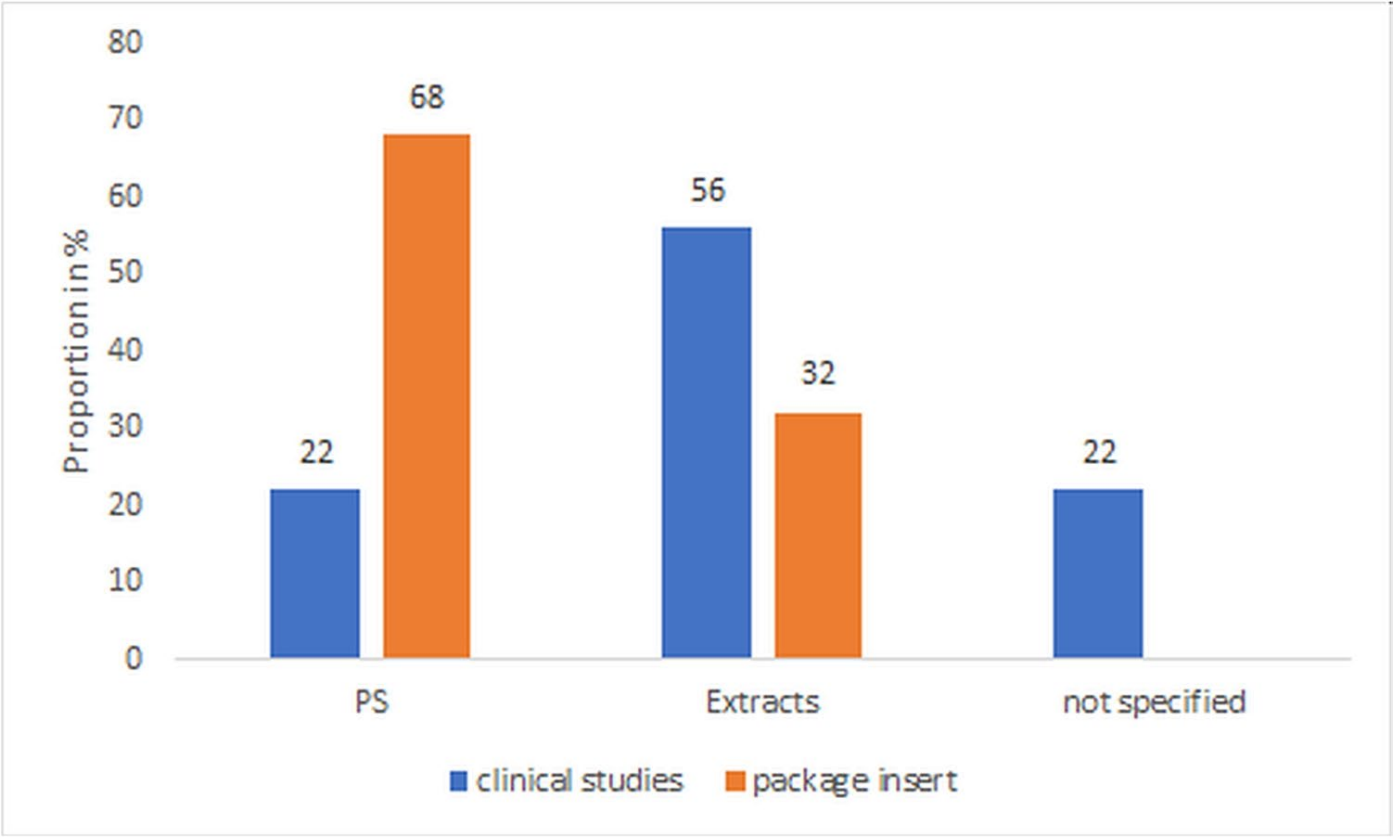
Comparison of the preparations selected in clinical studies with the preparations available on the market in percent

PS are the predominant type of preparation on the market. Stabilization is usually carried out with EtOH (Fig. 8). Extract preparations, mostly as EtOH extracts, follow in the frequency of products on the market. In 22% of the studies, the type of preparation was not specified. In contrast to the market products, extracts are predominantly used in the clinical studies, of which around half are ethanol-based. These are fundamentally different products from PS in terms of the ingredients; they contain. No conclusions can therefore be drawn from these studies about the efficacy and safety of the preparations on the market. It is therefore only possible to transfer the study situation to the market situation to a very limited extent.

Oral solutions represent half of the galenic dosage forms of the market preparations which corresponds to the study situation (Figure S11 Supplement). Tablets also account for a significant market share of 23%, while capsules and other dosage forms are represented to a lesser extent. In the clinical studies, tablets and capsules were in second place.

As the bioavailability can diverge due to different forms of administration, the various galenics should also be specifically investigated in this respect. After oral application of a tincture or tablets with an alkamide concentration of 0.07 mg each, the alkamides from the tincture were detectable after 30 min at 0.4 ng/ml and from the tablets after 45 min at 0.12 ng/ml serum (Woelkart et al. 2006). On the other hand, in another study, no differences were found in the serum uptake when using tincture or tablets (Matthias et al. 2007).

**Fig. 9:**
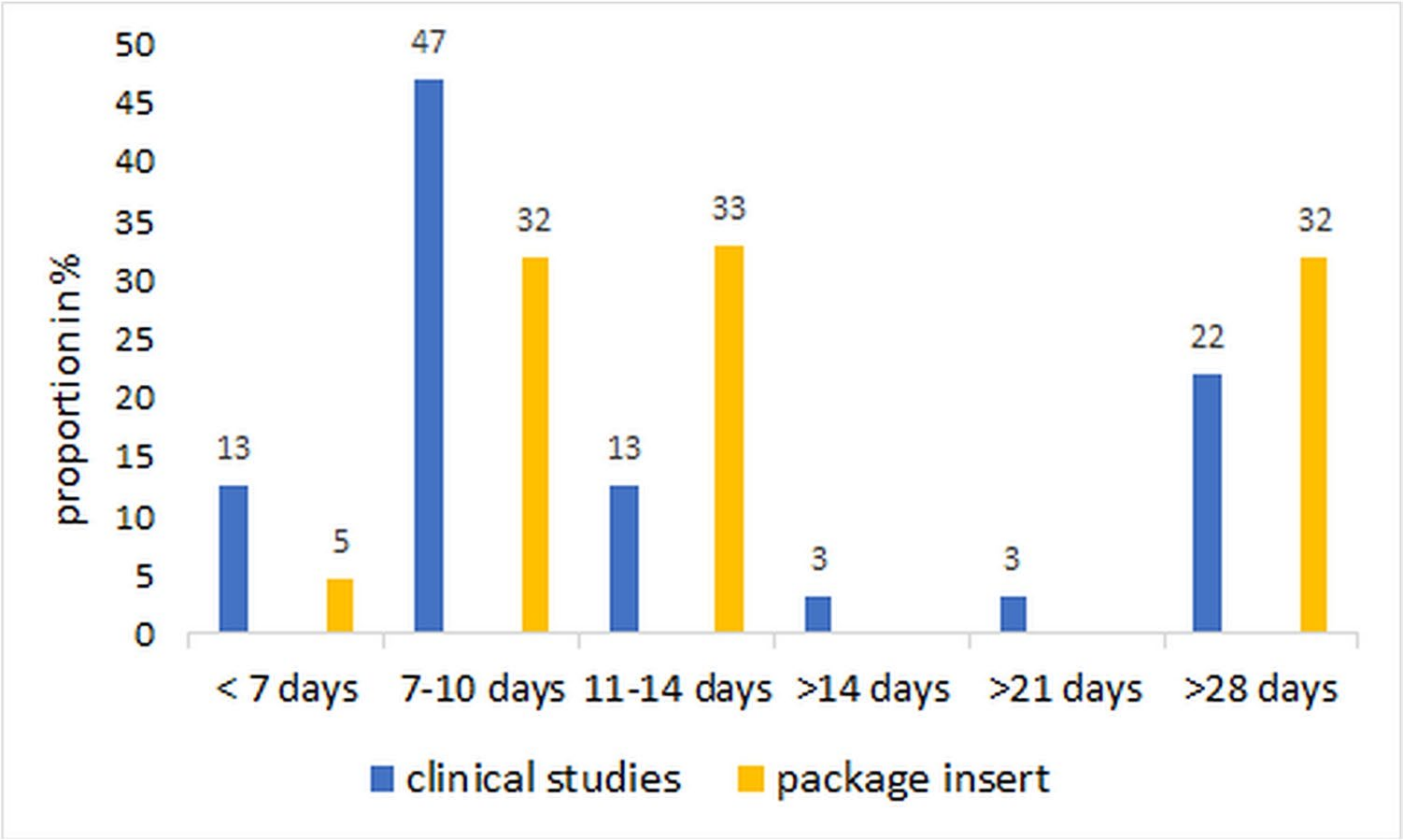
Comparison of the duration of administration of the preparations selected in clinical studies with the recommended duration of administration on the market

The recommended duration of administration for the market preparations varies, mostly 7–10, 11–14, or over 28 days are recommended (Fig. 9). In the studies, the intake was mostly for 7–10 days. The duration of use of 11–14 days is less frequently represented in studies. A duration of use of more than 28 days can be found with 22%. Long-term use in clinical trials is linked to studies on the prevention of *Echinacea.* In the case of market products, these are recommended by the manufacturer for prevention. Thus, the trend in the studies is towards a shorter duration of administration than declared on the market. Here, effects as well as interactions and adverse drug reactions could be overlooked.

**Fig. 10:**
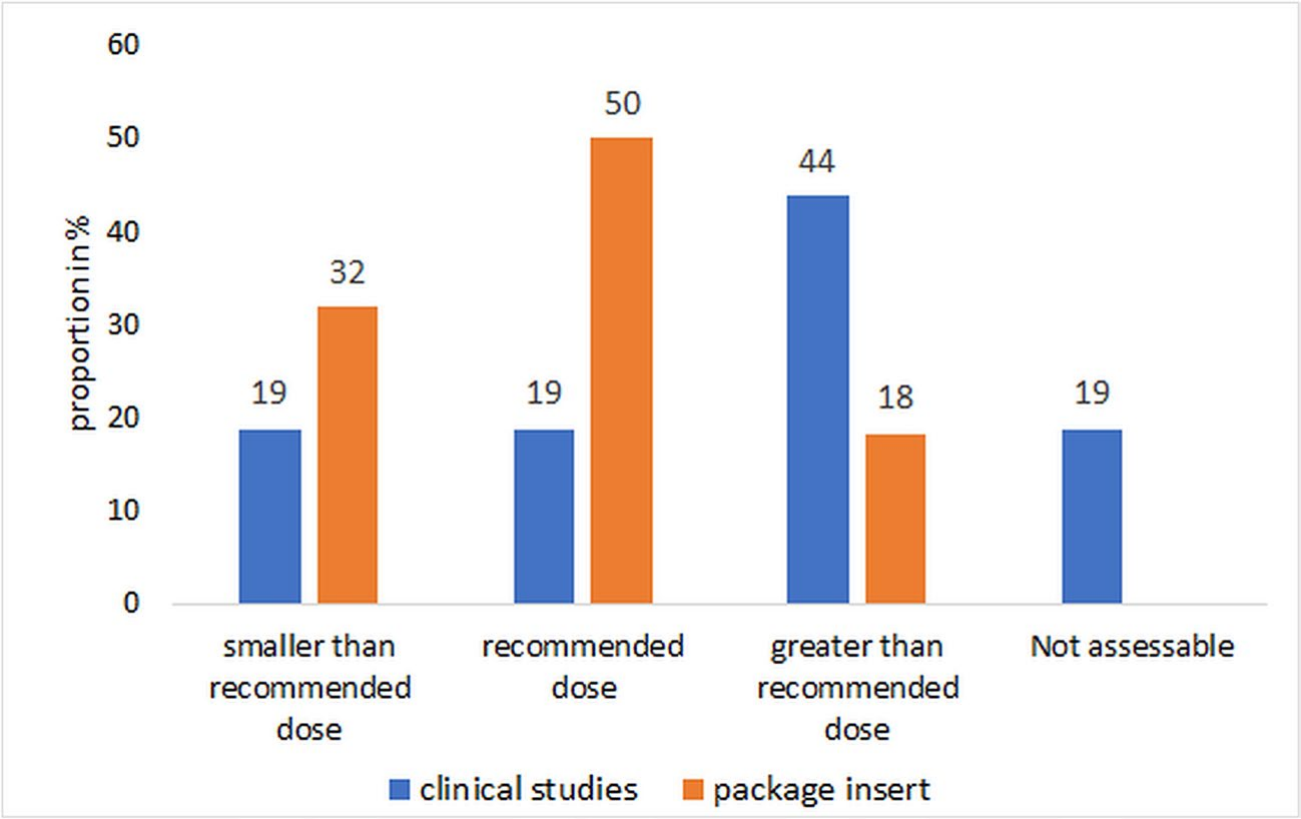
Comparison of the daily dose of the preparations selected in clinical studies with the daily dose recommended on the market. Recommended dose (Dingermann 2000): *E. purpurea* herba < 6–9 ml PS/ < 256–354 mg dried PS; *E. pallida* radix < 100–170 mg dry extract/ < 4.5–6 g tincture

There are no sufficient dose–response studies for the *Echinacea* preparations. For a comparative overview, the dose information in Dingermann (2000) was used and the doses used were classified accordingly. This had to be done as a guide, as the data did not always match exactly, and the information in the studies is not always clear. Half of the market preparations estimate a daily dose corresponding to the recommended daily dose (Fig. 10). Thirty-two percent of the market preparations are underdosed according to the declaration, and few of the market preparations declare an increased dosage. In the clinical studies, higher doses than recommended were predominantly used in comparison and in some cases, no assessable information on dosage was provided. If a higher dose than recommended was used in the studies, this may be positive with regard to the detection of ADRs.

**Fig. 11:**
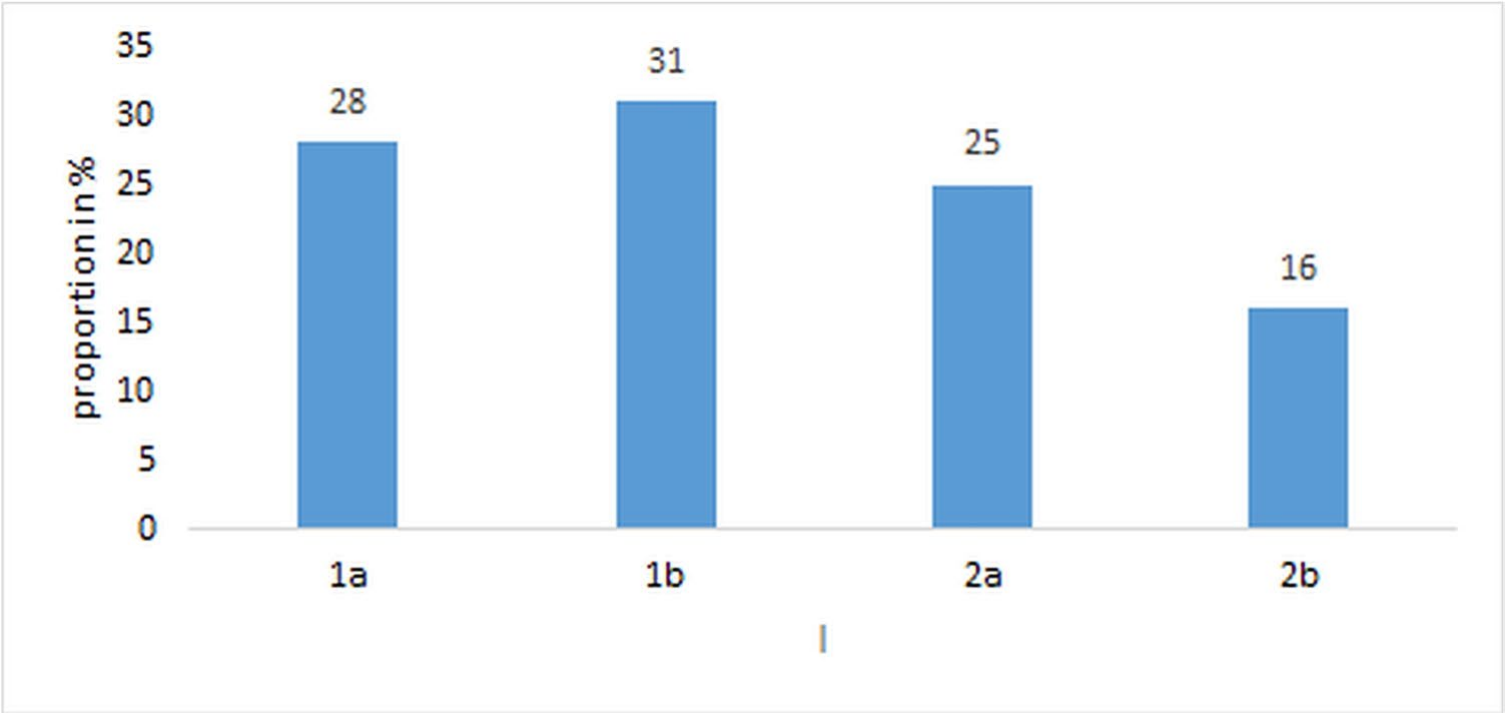
Type of clinical trials and selection of participants. 1: Treatment studies: a, participants suffering from the common cold; b, healthy participants. 2: Prevention studies: a, healthy participants (possibly susceptible to a cold); b, healthy participants artificially infected with *RV* 23 or 39

With regard to study conduct (Fig. 11), the clinical studies can be divided into treatment studies (participants with symptoms of a cold or healthy participants who started treatment at the onset of a cold) and prevention studies (healthy individuals, possibly susceptible to a cold or artificially infected with *RV* 23 or 39). Except for artificial infections, the cause of the cold symptoms was not investigated in any case. The assessment was usually based on a catalog of symptoms and only in a few cases with accompanying tests in blood and/or saliva (Figure S12 Supplement). In 53% of the studies, the symptoms were recorded by the participants themselves, which means that the objectivity of the assessment must remain unclear in individual cases. Data collection was carried out by medical staff and the participants in 34% of cases and by medical staff in a further 13% of cases (Figure S13 Supplement). Overall, 53% of the examinations showed a positive trend (44% treatment and 9% prevention studies), and no effect was found in 42% of the studies (13% for treatment and 29% for prevention studies). In particular, in the case of artificial infections with *RV*, no case of positive results could be found. Effects were not specified in 6% of the studies.

The variation within the clinical studies is large for all the criteria examined, making it almost impossible to compare and draw conclusions about the efficacy of *Echinacea* preparations. For future studies, standardization of the study conditions and a clear description of the preparations used would be important in order to be able to clearly assess efficacy. A dose study would also be useful to assess efficacy.

#### Studies vs. market situation — reference to the ingredients

Even if a reference to the ingredients is not required in the context of approvals and registrations, this aspect was analyzed. The in-process control during the manufacture of *Echinacea* preparations cannot be based on efficacy-determining substances, as the efficacy-determining substances have not yet been determined with certainty. Batch control is therefore carried out via betaine or Fructofuranosides (Loew et al. 1999; Information from manufacturers). However, this also means that a possible outcome could only be measured in clinical studies, as there are no other parameters for determining efficacy. If it would be possible to refer to certain ingredients with regard to the effect, a new method of standardization would not be transferable to preparations that are covered by the existing protection (Merz et al. 2016). In this context, *Echinacea* is and remains a product of the second half of the last century.

Standardization to process-sensitive substances was reported in 3% of the studies, and to one or more marker substances in 28% of the cases. No information was found in 69% of the studies. No information on standardization was provided in any of the package inserts. Approximately one-third of the preparations have at least been shown to contain certain substances, while the rest has not been tested in advance. The composition of the products on the market with regard to the ingredients is mostly unknown. Data was only available for five preparations that had been analyzed in a study. A further difficulty is that different batches of a product can also be very different (Bauer 1999).

### Limitations of the study

The data on the ingredients “plant species and part” for the products on the market were taken exclusively from the package inserts, because the availability of information for the customer should be examined. In some studies, the information on the methodology was not complete, but this is relevant to the analysis in terms of the resulting inability to classify the products for clinical use.

Little information on sales figures could be obtained, but this would have clarified the market relevance. The only information available is that 240 thousand packs of *Echinacea* monopreparations were sold in Offizina-pharmacies in 2022 with end consumer sales of approx. 2.8 million euros. This data cannot be used meaningfully, as there were certainly distortions in this market segment during this period due to the coronavirus pandemic and mail-order sales should also not be neglected.

## Conclusion

There is a large variety of *Echinacea* products on the market, all of which have been authorized or registered under pharmaceutical law — most of them for the first time long before the HMPC monographs were produced. The respective dried plant part is considered a drug in its entirety. Nevertheless, the products on the market can differ significantly from one another in terms of the composition of the ingredients in the end product, depending on the starting material and manufacturing process. Furthermore, the recommendations for use diverge — a uniform and reliable effect as well as a comparable effect when using different products is therefore not to be expected by the consumer. This is in contrast to the obvious homogeneity and the declared similar effect in the presentation of the products.

Antiviral, antibacterial, and antifungal as well as immunomodulatory effects were observed in vitro and in vivo, but these are of questionable clinical relevance due to the use of different preparations/only individual ingredients, different application methods (application partly intraperitoneal/intravenous), and high application doses. The low bioavailability of individual ingredients per os in connection with the high effect concentrations in the studies is not comparable with clinical use.

Alkamides, caffeic acid derivatives, and polysaccharides are found in the literature as marker substances for the effect of *Echinacea*. These also represent hydrophilic, hydrophobic, and polymeric substances, which enter the products to varying degrees depending on the type of plant preparation. Alkamides were detectable in the blood serum after ingestion and would therefore be bioavailable. Another possibility for the effectiveness of *Echinacea* is the direct contact in the throat via the epithelium during ingestion of the preparation. This effect was observed in vitro and could also be suspected in two clinical studies, in which the preparation was kept in the mouth for a short time after ingestion. This could confirm the effect observed in vitro that the antiviral effect only occurred in direct contact with the viruses in question. It does not appear to be effective against rhinoviruses that cause colds. The antiviral effect is primarily directed against enveloped viruses.

The comparison of the preparations used in the studies with the preparations available on the market showed a hiatus, and in some cases, the studies did not provide sufficient information on the preparation used. In some of the clinical studies, low dosages and shorter intake periods are used, and extracts are used in many studies, while pressed juices dominate the market. It must therefore also be questioned here whether the clinical studies reflect the clinical application. The study situation does not adequately reflect the market situation.

In future studies, the study population should reflect the patient population, and, if necessary, the outcome should be made more relevant. In connection with the great economic importance of colds mentioned above, the days of incapacity for work would be a suitable outcome parameter. In studies, the basic inclusion of specific hematological and biochemical measurement parameters could possibly improve the basis for assessment in addition to the assessment of symptoms by the participants. An analysis of preparations used in studies prior to their use in trials and a standardization of the study design are also part of a further approach for a safe and consistently effective use for *Echinacea* products.

At present, all *Echinacea* products on the market are legally compliant and have been described as phytotherapeutic agents in the course of the approval and registration procedures with regard to starting material and preparation methods. However, the products can still be very different in terms of their ingredients. The results of the available studies do not provide a clear statement on the efficacy of *Echinacea* and can only be applied to the products on the market to a very limited extent. This is not clear to customers from the package leaflets.

## Supplementary Information

Below is the link to the electronic supplementary material.

Supplementary file1 (DOCX 1437 KB)

## Data Availability

All source data for this work are available upon reasonable request.

## Abbreviations

ADR: Adverse drug reaction
ATC-Code: Anatomical therapeutic chemical (ATC) classification
A. Vogel: Alfred Max Vogel
AWI: Airway infection
CHMP: Committee for Medicinal Products for Human Use
COX: Cyclooxygenase
DAK: German employees health insurance
DER: Drug extract ratio
*E.*: *Echinacea*
ESCOP: European Scientific Cooperative on Phytotherapy
EU: European Union
GI: Gastrointestinal
HMPC: Committee on Herbal Medicinal Products
EU: European Union
LOX: Lipoxygenase
LURI: Lower urinary tract infection
NDR: North German Broadcasting
*RV*: Rhinovirus
*SARS-CoV*: Severe acute respiratory syndrome coronavirus
EtOH: Ethanol
PS: Pressed juice

## References

[CR2] Bauer R (1998) *Echinacea:* biological effects and active principles. Phytomedicines of Europe, American chemical society, Washington DC: 140–157. 10.1021/bk-1998-0691.ch02

[CR3] Bauer R (1999) Chemistry, analysis and immunological investigations of *Echinacea* phytopharmaceuticals. Immunmodulatory Agents from Plants 41–88. 10.1007/978-3-0348-8763-2_2

[CR4] Benson JM, Pokorny AJ, Rhule A, Wenner CA, Kandhi V, Cech NB, Shepherd DM (2010) *Echinacea* pupurea extracts modulate murine dendritic cell fate and function. Food Chem Toxicol 48(5):1170–1177. 10.1016/j.fct.2010.02.007 20149833 10.1016/j.fct.2010.02.007 PMC2883451

[CR5] Bergeron C, Gafner S (2007) Quantitative analysis of the polysaccharide and glycoprotein fractions in *Echinacea purpurea* and *Echinacea angustifolia*. by HPLC-ELSD for Quality Control of Raw Material. Pharm Biol 45(2), 98–105. 10.1080/13880200601112893

[CR7] Bodinet C, Lindequist U, Teuscher E, Freudenstein J (2004) Influence of peroral application of a herbal immunomodulator on the antibody production of Peyer’s patches cells. Arzneimittelforschung 54(2):114–118. 10.1055/s-0031-1296945 15038461 10.1055/s-0031-1296945

[CR8] Budinger V (2020) Run auf Schweizer Echinacea-Extrakt. Eine fehlgedeutete In-vitro-Studie erzeugt falsche Hoffnungen im Kampf gegen COVID-19. DAZ 39, 26. https://www.deutsche-apotheker-zeitung.de/daz-az/2020/daz-39-2020/run-auf-schweizer-echinacea-extrakt. Abgerufen am 05 Juli 2023.

[CR9] Burger RA, Torres AR, Warren RP, Caldwell VD, Hughes BG (1997) *Echinacea*-induced cytokine production by human macrophages. Int J Immunopharmacol 19(7):371–378. 10.1016/s0192-0561(97)00061-1 9568541 10.1016/s0192-0561(97)00061-1

[CR10] Claasen B (2018) Arabinogalaktan-Proteine (AGPs) aus dem kurzzeiterhitzten Presssaft von *Echinacea purpurea*. Zeitschrift Für Phytotherapie 39(04):152–158. 10.1055/a-0636-1977

[CR11] Classen B, Pferschy-Wenzig E-M, Geske T, Ardjomand-Wölkart K, Bauer R (2019) (2019) Analytische Charakterisierung und Vergleich medizinisch genutzter *Echinacea*-haltiger Zubereitungen. Zeitschrift Für Phytotherapie 40(04):148–157. 10.1055/a-0843-1655

[CR12] Dingermann T (2000) (Hrsg.): Transparenzkriterien für pflanzliche, homöopathische und anthroposophische Arzneimittel. Basel, Freiburg, Paris. S. Karger

[CR13] ESCOP Monographs Online-series (2021) *Echinaceae purpureae* herba (Purple Coneflower Herb). https://www.escop.com/downloads/echinaceae-purpureae-herba-purple-coneflower-herb/

[CR16] European Medicines Agency (EMA/HMPC) (2023a) *Echinaceae purpureae* herba recens – herbal medicinal product. Purple Coneflower herb.( EMA/HMPC/48704/2014 Corr.; EMA/HMPC/557979/2013, Committee on Herbal Medicinal Products (HMPC)). https://www.ema.europa.eu/en/medicines/herbal/echinaceae-purpureae-herba. Abgerufen am 4 Juli 2023

[CR14] European Medicines Agency (EMA/HMPC) (2023b) *Echinaceae purpureae* radix - herbal medicinal product. Purple Coneflower Root. EMA/HMPC/424583/2016; EMA/HMPC/424584/2016, Committee on Herbal Medicinal Products (HMPC)). https://www.ema.europa.eu/en/medicines/herbal/echinaceae-purpureae-radix. Abgerufen am 4 Juli 2023

[CR15] European Medicines Agency (EMA/HMPC) (2023c) *Echinaceae pallidae* radix - herbal medicinal product. Pale Coneflower Root. (EMA/HMPC/737380/2018; EMA/HMPC/737379/2017, Committee on Herbal Medicinal Products (HMPC)). https://www.ema.europa.eu/en/medicines/herbal/echinaceae-pallidae-radix. Abgerufen am 4 Juli 2023

[CR17] European pharmacopoeia (2020) 10th edition, basic work Deutscher Apothekerverlag

[CR19] Gilroy CM, Steiner JF, Byers T, Shapiro H, Georgian W (2003) Echinacea and truth in labeling. Arch Inter Med 163(6):699–704. 10.1001/archinte.163.6.699 10.1001/archinte.163.6.699 12639203

[CR20] Graefe E, Veit M (1999) Urinary metabolites of flavonoids and hydroxycinnamic acids in humans after application of a crude extract from *Equisetum arvense*. Phytomedicine 6(4):239–246. 10.1016/S0944-7113(99)80015-4 10589442 10.1016/S0944-7113(99)80015-4

[CR21] health.ec.europa.eu (2001a) Directive 2001/83/EC of the European Parliament and of the council of 6 November 2001 on the community code relating to medicinal products for human use. Article 10a of Directive 2001/83/EC. https://health.ec.europa.eu/document/download/6a59e03f-fb86-4cbc-9fca-f8e4a7e938b1_en?filename=dir_2001_83_cons_2012_en.pdf. Abgerufen am 5. Oktober 2023

[CR22] health.ec.europa.eu (2001b) Directive 2001/83/EC of the European parliament and of the council of 6 November 2001 on the community code relating to medicinal products for human use. Article 16a(1) of Directive 2001/83/EC. https://health.ec.europa.eu/document/download/6a59e03f-fb86-4cbc-9fca-f8e4a7e938b1_en?filename=dir_2001_83_cons_2012_en.pdf. Abgerufen am 5 Oktober 2023

[CR24] Hudson J, Vimalanathan S, Kang L, Amiguet VT, Livesey J, Arnason JT (2005) Characterization of antiviral activities in *Echinacea* root preparations. Pharm Biol 43(9):790–796. 10.1080/13880200500408491

[CR25] Jager H, Meinel L, Dietz B, Lapke C, Bauer R, Merkle HP, Heilmann J (2002) Transport of alkamides from *Echinacea* species through Caco-2 monolayers. Planta Med 68(5):469–471. 10.1055/s-2002-32076 12058332 10.1055/s-2002-32076

[CR26] Karsch-Völk M, Barrett B, Kiefer D, Bauer R, Ardjomand-Woelkart K, Linde K (2014) *Echinacea* for preventing and treating the common cold (review). Cochrane Library. 10.1002/14651858.CD000530.pub3 10.1002/14651858.CD000530.pub3 PMC4068831 24554461

[CR27] Kolev E, Mircheva L, Edwards MR, Johnston SL, Kalinov K, Stange R, Gancitano G, Vanden Berghe W, Kreft S (2022) *Echinacea Purpurea* for the long-term prevention of viral respiratory tract infections during Covid-19 pandemic: a randomized, open, controlled, exploratory clinical study. Front Pharmacol 13:856410–856419. 10.3389/fphar.2022.856410 35559249 10.3389/fphar.2022.856410 PMC9087554

[CR28] . Liu Y, Murphy PA (2007) Alkamide stability in *Echinacea purpurea* extracts with and without phenolic acids in dry films and in solution. J Agric Food Chem. 10;55(1):120–126. 10.1021/jf0619481 10.1021/jf0619481 PMC1964881 17199322

[CR29] Loew D, Blume H, Dingermann Th (1999) (Hrsg.) unter Mitarbeit von Schubert-Zsilavecz M.: Phytopharmaka V. Forschung und klinische Anwendung. Darmstadt Steinkopff. 10.1007/978-3-642-58709-2

[CR30] Luo XB, Chen B, Yao SZ, Zeng JG (2003) Simultaneous analysis of caffeic acid derivatives and alkamides in roots and extracts of *Echinacea purpurea* by high-performance liquid chromatography-photodiode array detection-electrospray mass spectrometry. J Chromatogr A. 31;986(1):73–81. 10.1016/s0021-9673(02)01922-2 10.1016/s0021-9673(02)01922-2 12585324

[CR31] Maass N, Bauer J, Paulicks BR, Böhmer BM, Roth-Maier DA (2005) Efficiency of *Echinacea purpurea* on performance and immune status in pigs. J Anim Physiol Anim Nutr 89:244–252. 10.1111/j.1439-0396.2005.00501.x 10.1111/j.1439-0396.2005.00501.x 15972074

[CR32] Matthias A, Penman KG, Matovic NJ, Bone KM, De Voss JJ, Lehmann RP (2005) Bioavailability of *Echinacea* constituents: Caco-2 monolayers and pharmacokinetics of the alkylamides and caffeic acid conjugates. Molecules 10(10):1242–1251. 10.3390/10101242 18007516 10.3390/10101242 PMC6147618

[CR33] Matthias A, Addison RS, Agnew LL, Bone KM, Watson K, Lehmann RP (2007) Comparisdon of *Echinacea* alkylamide pharmokonkinectics between liquid and table preparations. Phytomedicine 14(9):587–590. 10.1016/j.phymed.2006.12.021 17289362 10.1016/j.phymed.2006.12.021

[CR34] Merz B, Flemisch S, Wiesner J, Knöss W: Der wissenschaftliche Kenntnisstand zu Ginkgoblättern (*Ginkgo biloba* L., folium) und Zubereitungen. Bulletin zur Arzneimittelsicherheit (2016). Informationen aus BfArM und PEI. Ausgabe 4, 6–10. https://www.pei.de/SharedDocs/Downloads/DE/newsroom/bulletin-arzneimittelsicherheit/2016/4-2016.pdf?__blob=publicationFile&v=2. Abgerufen am 3 Oktober

[CR35] NDR (2023) Erkältung mit Husten und Schnupfen : Diese Hausmittel helfen. https://www.ndr.de/ratgeber/gesundheit/Erkaeltung-mit-Husten-und-Schnupfen-Diese-Hausmittel-helfen,erkaeltung196.html. Abgerufen am 2 März 2024

[CR36] Nicolussi S, Ardjomand-Woelkart K, Stange R, Gancitano G, Klein P, Ogal M (2022) *Echinacea* as a potential force against coronavirus infections? A mini-review of randomized controlled trials in adults and children. Microorganisms 10(2):211. 10.3390/microorganisms10020211 35208665 10.3390/microorganisms10020211 PMC8879308

[CR37] Nüsslein B, Kurzmann M, Bauer R, Kreis W (2000) Enzymatic degradation of cichoric acid in *Echinacea purpurea* preparations. J Nat Prod 63(12):1615–1618. 10.1021/np0002839) 11141099 10.1021/np0002839

[CR38] Öko-Test Jahrbuch Gesundheit für 2010 (2010). Echinaceapräparate Alter Hut!., 30 - 33. https://www.oekotest.de/static_files/pdfs/article/94022/download.pdf. Abgerufen am 2.06.2023

[CR39] Osowski S, Rostock M, Bartsch H-H, Massing U (2000) Zur pharmazeutischen Vergleichbarkeit von therapeutisch verwendeten *Echinacea*-Präparaten. Complement Med Res 7(6):294–300. 10.1159/000057177 10.1159/000057177 11155023

[CR40] Perry NB, van Klink JW, Burgess EJ, Parmenter GA (1997) Alkamide levels in *Echinacea purpurea*: a rapid analytical method revealing differences among roots, rhizomes, stems, leaves and flowers. Planta Med 63(01):58–62. 10.1055/s-2006-957605 17252329 10.1055/s-2006-957605

[CR41] Perry NB, Burgess EJ, Glennie VL (2001) *Echinacea* standardization: analytical methods for phenolic compounds and typical levels in medicinal species. J Agric Food Chem 49(4):1702–1706. 10.1021/jf001331y 11308313 10.1021/jf001331y

[CR42] Pleschka S, Stein M, Schoop R, Hudson JB (2009) Anti-viral properties and mode of action of standardizedEchinacea purpureaextract against highly pathogenic avian Influenza virus (H5N1, H7N7) and swine-origin H1N1 (S-OIV). Virol J 6(1):197–205. 10.1186/1743-422X-6-197 19912623 10.1186/1743-422X-6-197 PMC2785784

[CR43] Pugh ND, Balachandran P, Lata H, Dayan FE, Joshi V, Bedir E, Makino T, Moraes R, Khan I, Pasco DS (2005) Melanin: dietary mucosal immune modulator from *Echinacea* and other botanical supplements. Int Immunopharmacol 5(4):637–647. 10.1016/j.intimp.2004.12.011 15710333 10.1016/j.intimp.2004.12.011

[CR44] Randolph RK, Gellenbeck K, Stonebrook K, Brovelli E, Qian Y, Bankaitis-Davis D, Cheronis J (2003) Regulation of human immune gene expression as influenced by a commercial blended *Echinacea* product: preliminary studies. Exp Biol Med 228(9):1051–1056. 10.1177/153537020322800910 10.1177/153537020322800910 14530514

[CR45] Roessler J, Steinmuller C, Kiderlen A, Emmendorffer A, Wagner H, Lohmann-Matthes ML (1991) Application of purified polysaccharides from cell cultures of the plant *Echinacea purpurea* to mice mediates protection against systemic infections with *Listeria monocytogenes* and *Candida albicans*. Int J Immunopharmacol 13(1):27–37. 10.1016/0192-0561(91)90022-y 2026472 10.1016/0192-0561(91)90022-y

[CR46] Rösch W. (2008) *Echinacea* bei Erkältungen wirksam. Dtsch Arztebl, 105(44), 766. https://www.aerzteblatt.de/archiv/62159/Echinacea-bei-Erkaeltungen-wirksam. Abgerufen am 3 Juli 2023

[CR48] Sharma M, Arnason JT, Burt A, Hudson JB (2006) Echinacea extracts modulate the pattern of chemokine and cytokine secretion in rhinovirus-infected and uninfected epithelial cells. Phytother Res 20:147–152. 10.1002/ptr.1824 16444669 10.1002/ptr.1824

[CR49] Signer J, Jonsdottir HR, Albrich WC, Strasser M, Züst R, Ryter S, Ackermann-Gäumann R, Lenz N, Siegrist D, Suter A, Schoop R, Engler OB (2020a) In vitro virucidal activity of Echinaforce®, an *Echinacea* purpurea preparation, against coronaviruses, including common cold coronavirus 229E and SARS-CoV-2. Virology J 17(1):136–146 32907596 10.1186/s12985-020-01401-2 PMC7479405

[CR51] Signer J, Jonsdottir HR, Albrich WC, Strasser M, Züst R, Ryter S, Ackermann-Gäumann R, Lenz N, Siegrist D, Suter A, Schoop R, Engler OB (2020b) Author correction: in vitro virucidal activity of Echinaforce®, an Echinacea purpurea preparation, against coronaviruses, including common cold coronavirus 229E and SARS-CoV-2. Virology Journal 17(1):172–175. 10.1186/s12985-020-01439-2 33168000 10.1186/s12985-020-01439-2 PMC7649903

[CR52] Simonetti P, Gardana C, Pietta P (2001) Plasma levels of caffeic acid and antioxidant status after red wine intake. J Agric Food Chem 49(12):5964–5968. 10.1021/jf010546k 11743793 10.1021/jf010546k

[CR53] Statista (2021) Meistverkaufte rezeptfreie apothekenpflichtige Arzneimittel Deutschlands. https://de.statista.com/page/otc. Abgerufen am 1 Juli 2023

[CR54] Steinhoff B (2008) Phytopharmaka in Europa. DAZ 30, 46. https://www.deutsche-apotheker-zeitung.de/daz-az/2008/daz-30-2008/phytopharmaka-in-europa. Abgerufen am 18 August 2023

[CR55] Trabert M, Seifert R (2023) Critical analysis of *gingko* preparations: comparison of approved drugs and dietary supplements marketed in Germany. Naunyn-Schmiedeberg’s Arch Pharmacol 397:451–461. 10.1007/s00210-023-02602-6 37470803 10.1007/s00210-023-02602-6 PMC10771617

[CR56] Uang YS, Hsu KY (1997) a dose-dependent pharmocokinetic study on caffeic acid in rabbits after intravenous administration. Biopharm Drug Dispos 18(8):727–736. 10.1002/(sici)1099-081x(199711)18:8%3c727::aid-bdd58%3e3.0.co;2-f 9373729 10.1002/(sici)1099-081x(199711)18:8<727::aid-bdd58>3.0.co;2-f

[CR58] Vimalanathan S, Arnason JT, Hudson JB (2009) Anti-inflammatory activities of *Echinacea* extracts do not correlate with traditional marker components. Pharm Biol 47(5):430–435. 10.1080/13880200902800204

[CR59] Vimalanathan S, Shehata M, Sadasivam K, Delbue S, Dolci M, Pariani E, D’Alessandro S, Pleschka S (2022) Broad antiviral effects of *Echinacea purpurea* against SARS-CoV-2 variants of concern and potential mechanism of action. Microorganisms 10(11):2145. 10.3390/microorganisms10112145 36363737 10.3390/microorganisms10112145 PMC9694187

[CR60] Voigt E (2024) Krankmeldungen im Job waren 2023 erneut auf Höchststand.ZEIT ONLINE. https://www.zeit.de/gesundheit/2024-01/krankschreibung-rekord-deutschland. Abgerufen am 28. März 2024

[CR61] Wang NG, Li YP, Yuan SK, Zhang H, Ren J, Ren X, Liu JX (2022) The intestinal absorption mechanism of chicoric acid and its bioavailability improvement with chitosan. Heliyon 8(7):e09955. 10.1016/heliyon.2022.e09955 35874082 10.1016/j.heliyon.2022.e09955 PMC9304723

[CR62] Wegner C (2022) Sars-CoV-2 : *Echinacea* reduziert Viruslast. Pharmacy adhoc. https://www.apotheke-adhoc.de/nachrichten/detail/pharmazie/sars-cov-2-echinacea-reduziert-viruslast-neue-daten-untermauern-wirksamkeit/. Abgerufen am 3 Juli 2023

[CR63] Woelkart K, Bauer R (2007) The role of alkamides as an active principle of *Echinacea*. Planta Med 73(7):615–623. 10.1055/s-2007-981531 17538868 10.1055/s-2007-981531

[CR64] Woelkart K, Marth E, Suter A, Schoop R, Raggam RB, Koidl C, Kleinhappl B, Bauer R (2006) Bioavailability and pharmacokinetics of *Echinacea purpurea* preparations and their interaction with the immune system. Int J Clin Pharmacol Ther 44(09):401–408. 10.5414/cpp44401 16995328 10.5414/cpp44401

