# Supplementary material for: Critical analysis of Echinacea preparations marketed in Germany
Source: Naunyn Schmiedebergs Arch Pharmacol. 2024 Nov 28;398(5):5743–56. doi: 10.1007/s00210-024-03634-2 (PMC11985562; doi:10.1007/s00210-024-03634-2)
Supplement: Supplementary file 1 — Supplementary file1 (DOCX 1437 KB) [file 210_2024_3634_MOESM1_ESM.docx]

**Critical analysis of *echinacea* preparations marketed in Germany**

**Carina Groß and Roland Seifert**

**Supplemental Information**

| **well-established use** | **traditional use** |
| --- | --- |
| Article 10a of Directive 2001/83/EC | Article 16a (1) of Directive 2001/83/EC |
| Medical use for at least 10 years in the EU and at least one high-quality, randomized-controlled clinical study has been published on efficacy and safety. | Medical use for at least 30 years, including at least 15 years in the EU. Efficacy and safety are therefore considered proven. Clinical studies are not required. |
| HMPC monograph for this use is available, then compliance must be shown:  - equivalent strength and dose  - same plant species  - same plant part  - same extractant in the same  concentration  - same or comparable DEV  - same form of preparation |  |

**Fig. S 1:** Comparison of the requirements for approval/registration in the “well-established
 and traditional use”

**Market analysi****s**

**
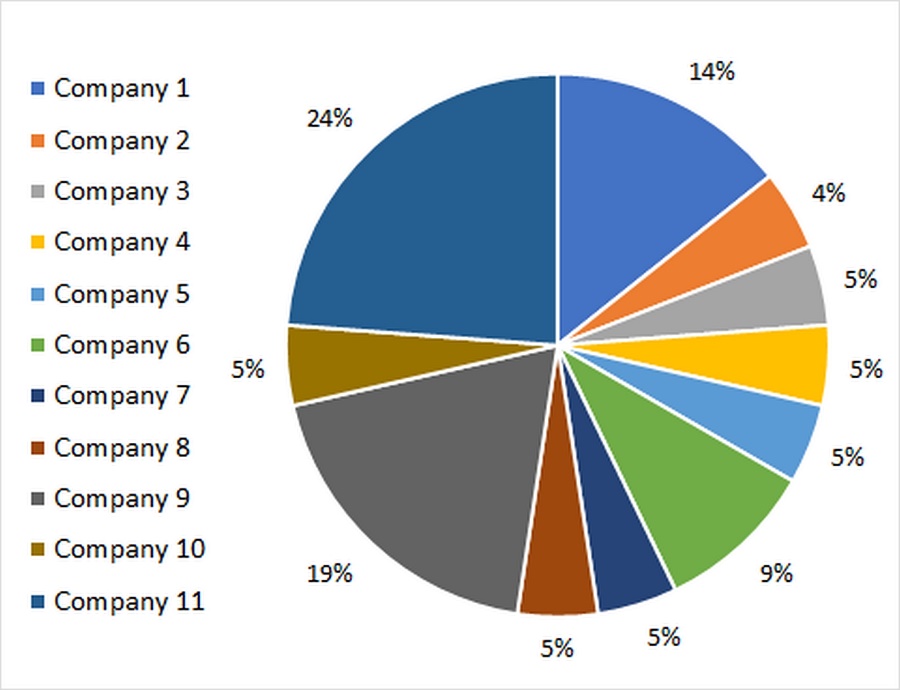
**

**Fig. S 2:** Marketing authorization holders of *Echinacea* monopreparations as a pie chart, the
 market shares of the marketing authorization holders are shown in color.


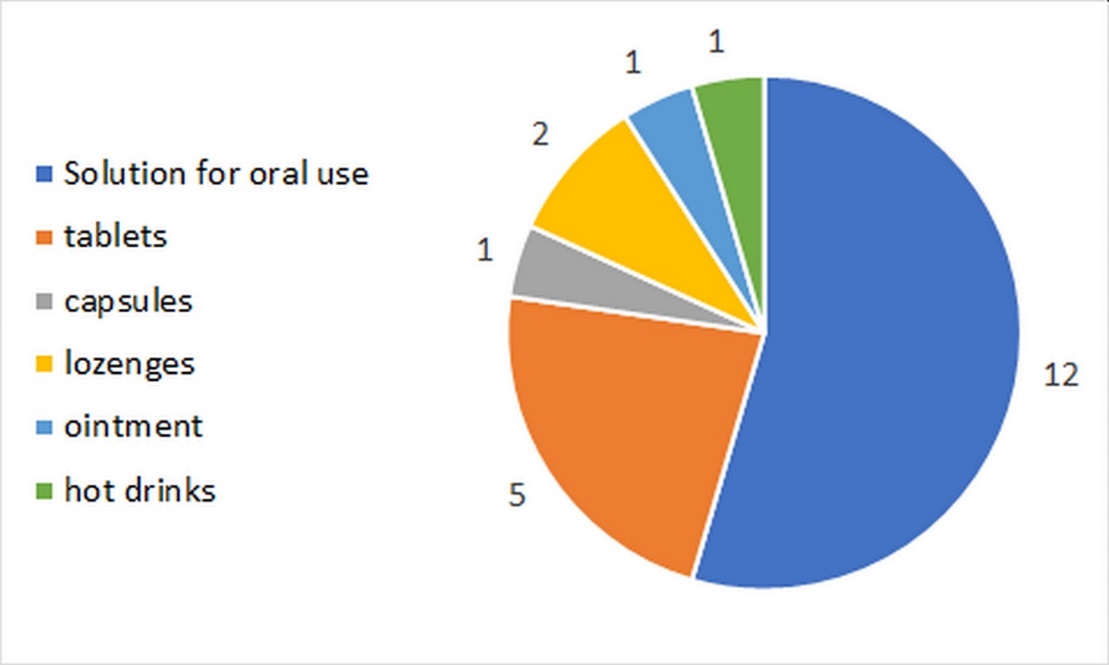


**Fig. S 3:** Absolute number of dosage forms of *Echinacea* monopreparations shown as a pie
 chart. The different dosage forms are shown in color.

**
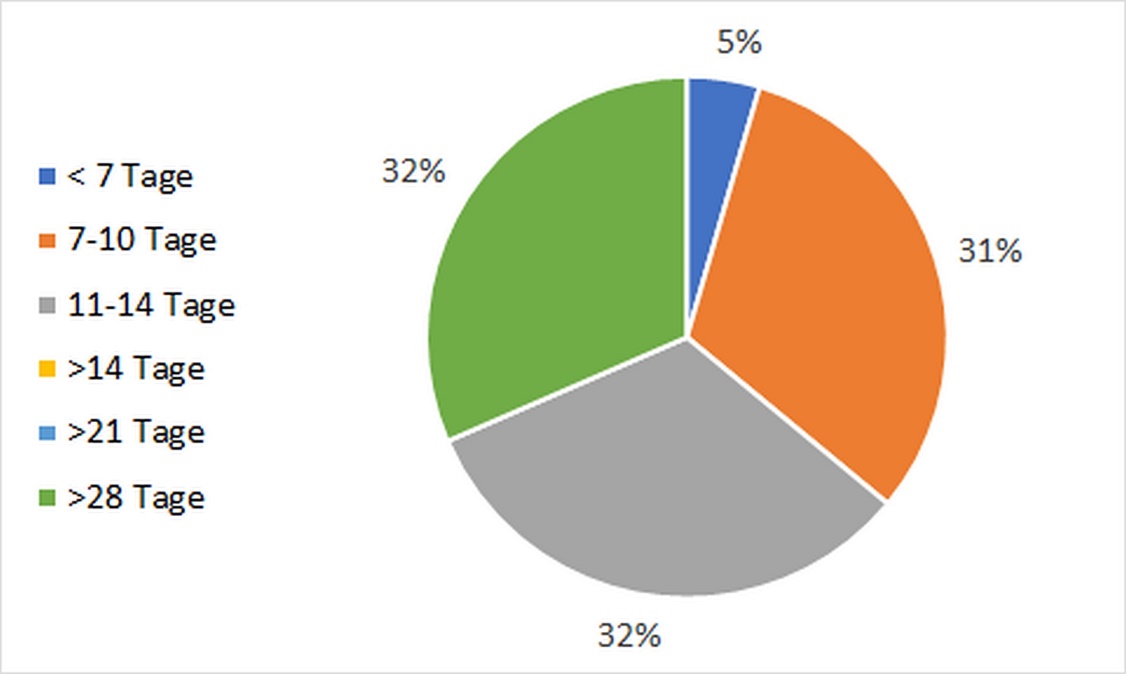
**

**Fig. S 4:** Recommended intake periods of *Echinacea* monopreparations plotted in color as a
 pie chart**.**

**
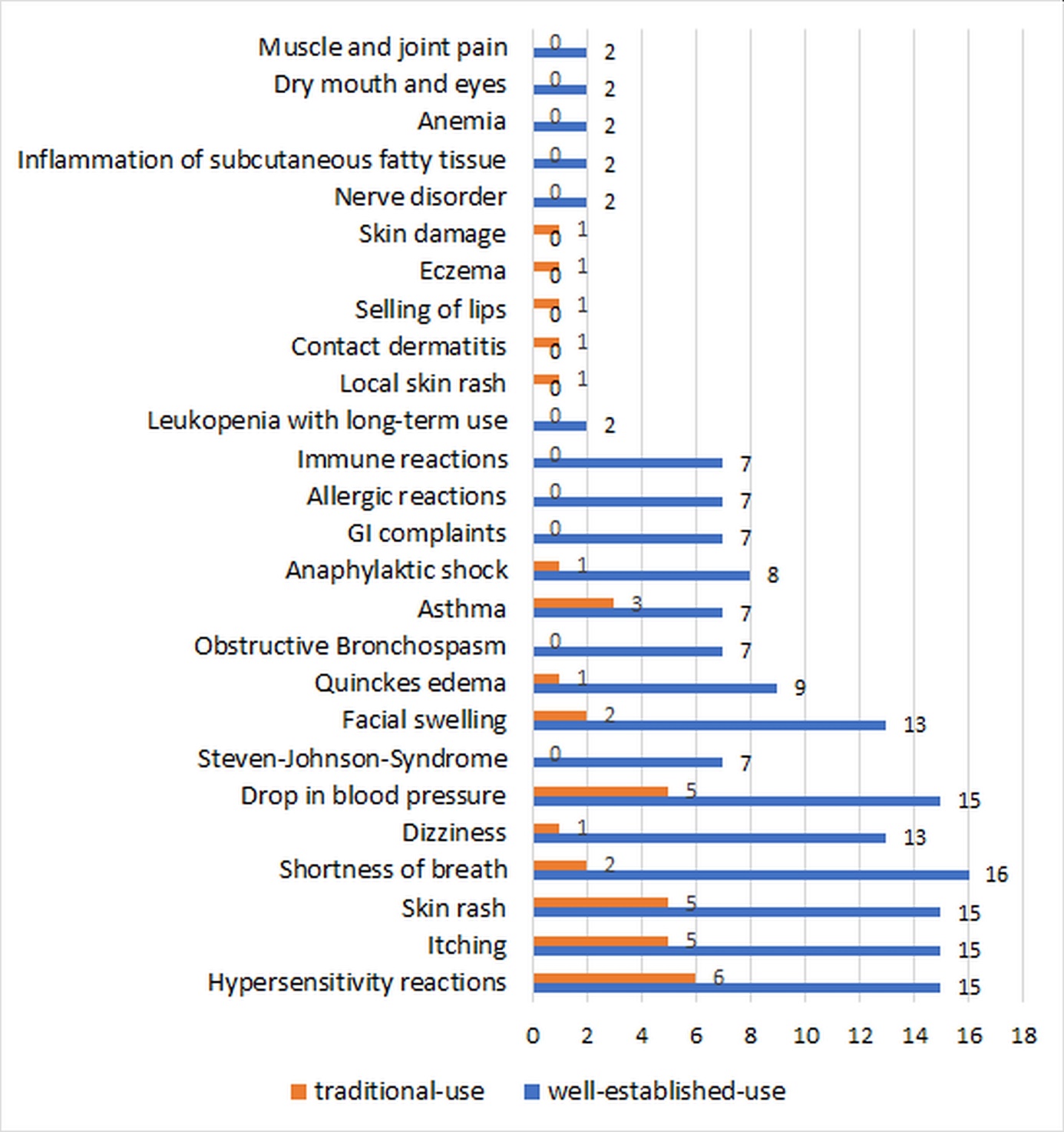
**

**Fig. S 5:** Absolute number of declared adverse drug reactions of *Echinacea*
 monopreparations by type of authorization/registration plotted as a bar chart.
 Registration in "traditional use" shown in orange and registration in "well
 established use" shown in blue


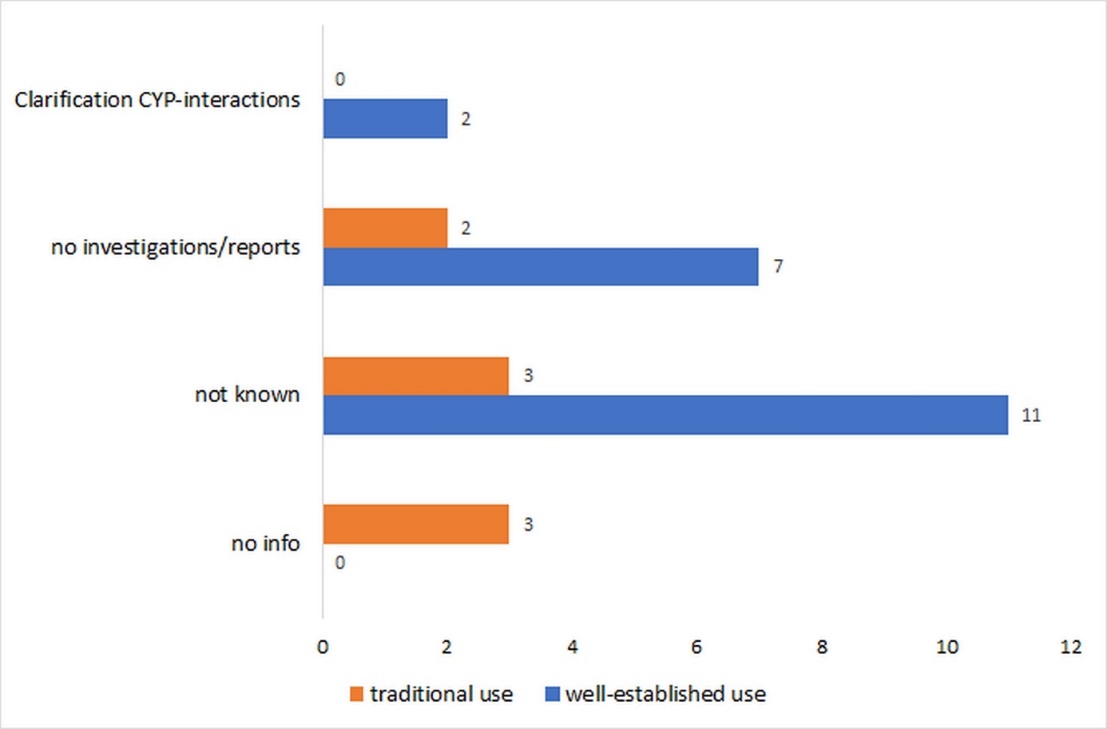


**Fig. S 6:** Absolute number of declared interactions of *Echinacea* monopreparations by type
 of authorization/registration plotted as a bar chart. Registration in "traditional use"
 shown in orange and registration in "well-established use" shown in blue

**
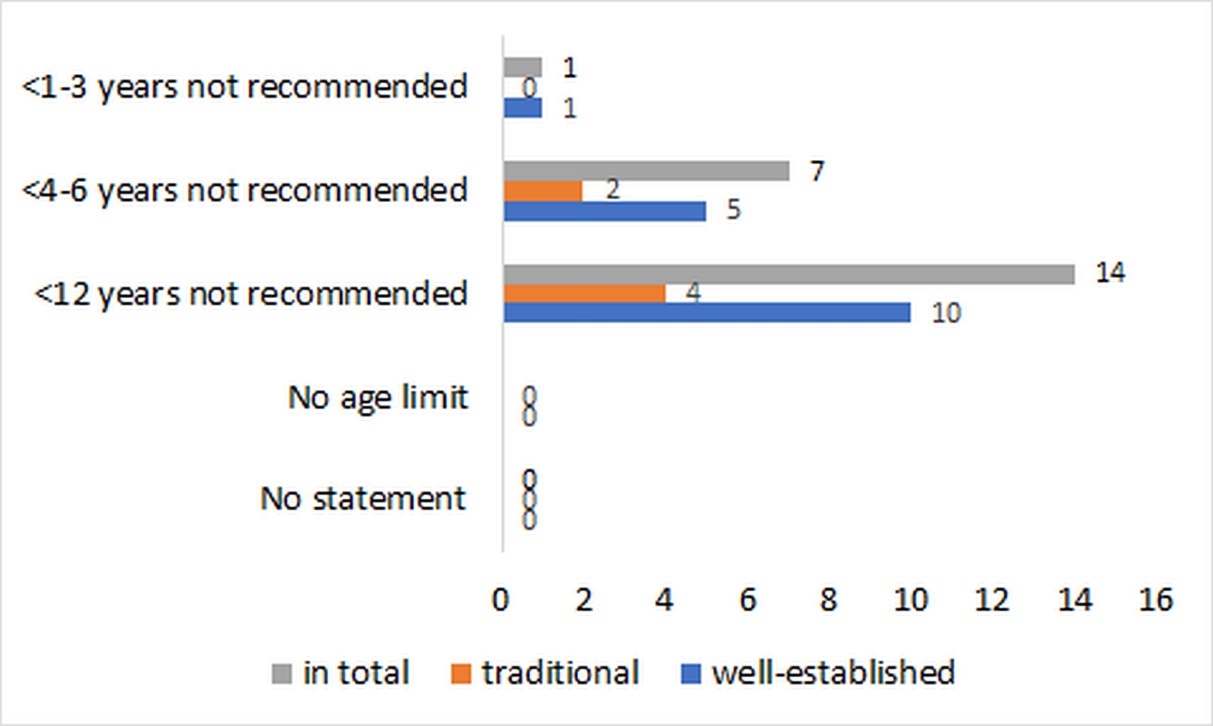
**

**Fig. S 7:** Absolute number of declared recommendations for the use of *Echinacea*
 monopreparations in children, plotted as a bar chart according to type of
 authorization/registration. Registration in "traditional use" orange, registration in
 "well-established use" blue and total number shown in green

**In vitro Studies**

The respective study design depended on the study objective, the preparations used and the target structures. Like the results, these were highly heterogeneous and therefore pooling was not possible. The following graphs summarize this**.**

**
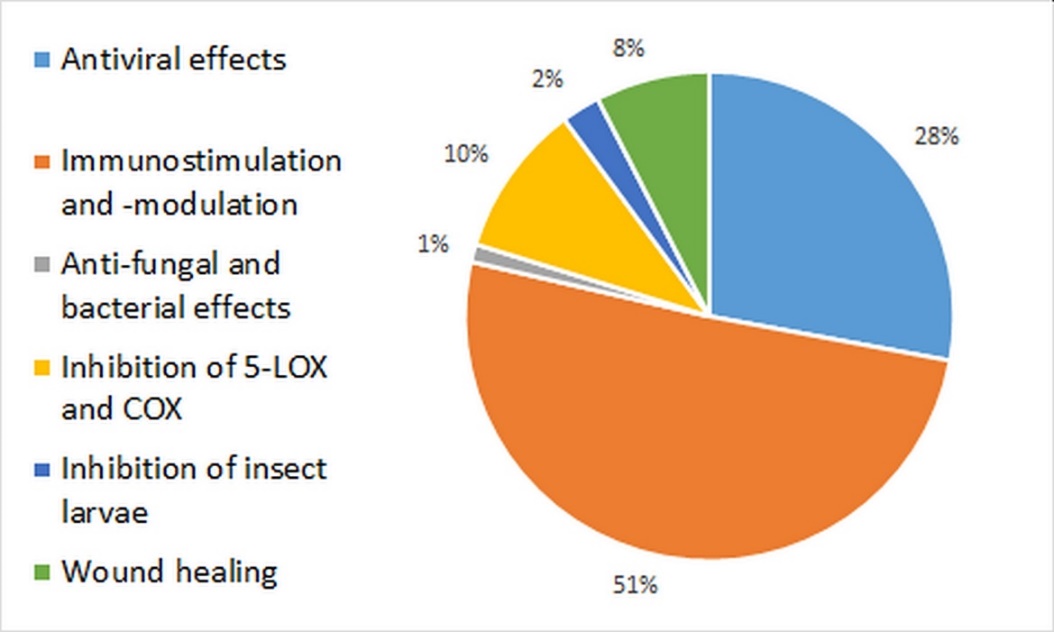
**

**Fig. S 8:** Analysis of the study objectives of the in vitro studies plotted in color as a pie chart


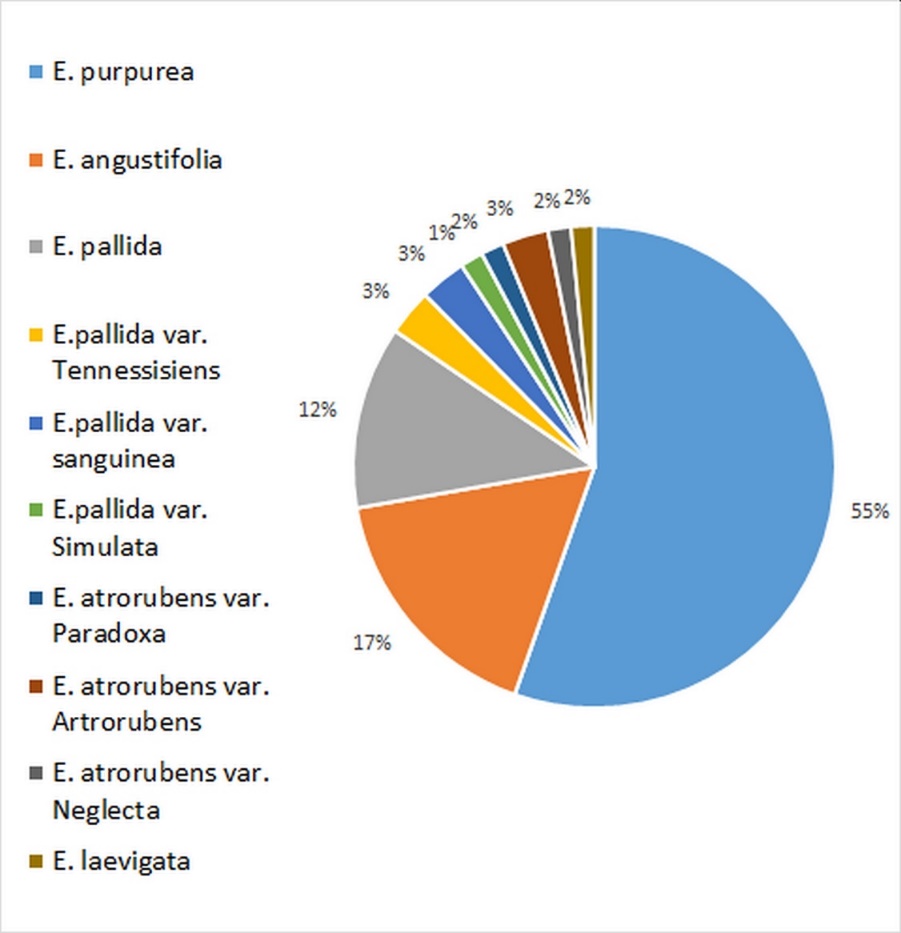


**Fig. S 9:** Analysis of the plant species used in in vitro studies plotted in color as a pie chart


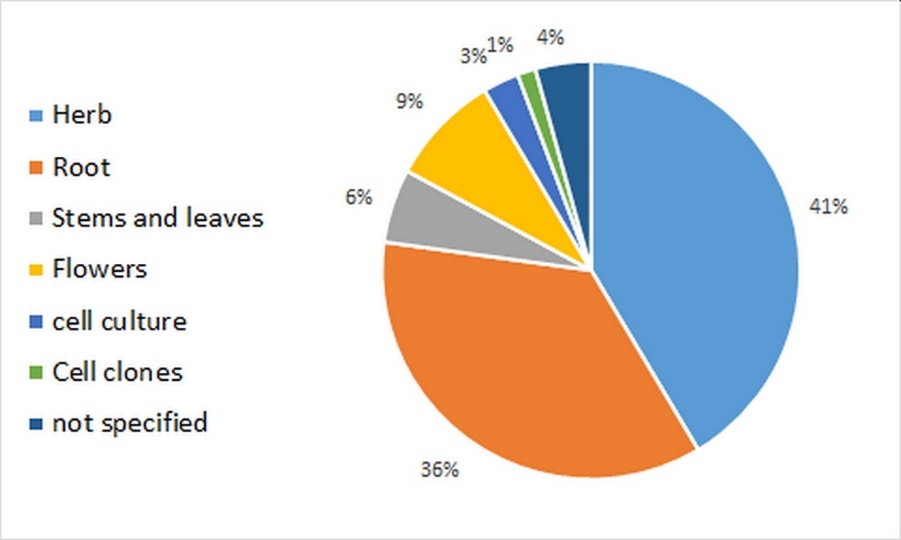


**Fig. S 10:** Plant parts used in in vitro studies plotted in color as a pie chart


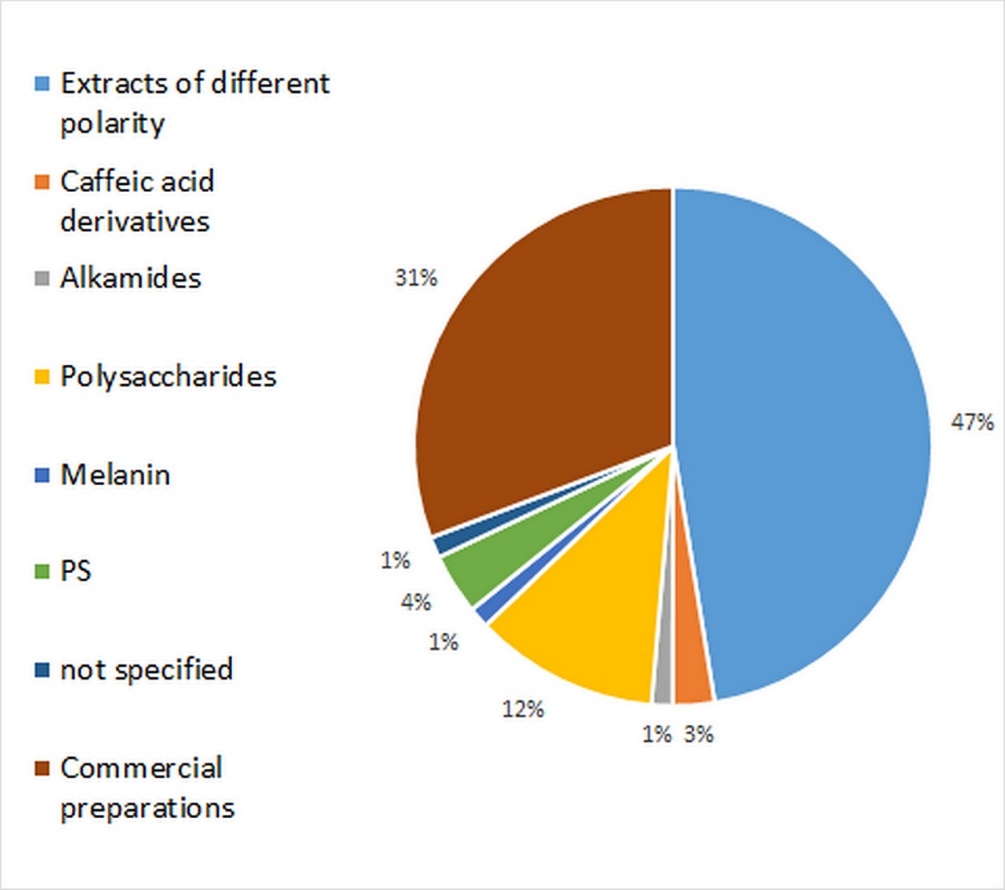


**Fig.S 11:** Analysis of the preparations and components used in in-vitro studies plotted in
 color as a pie chart

**Comparison clinical studies and preparations on the market**


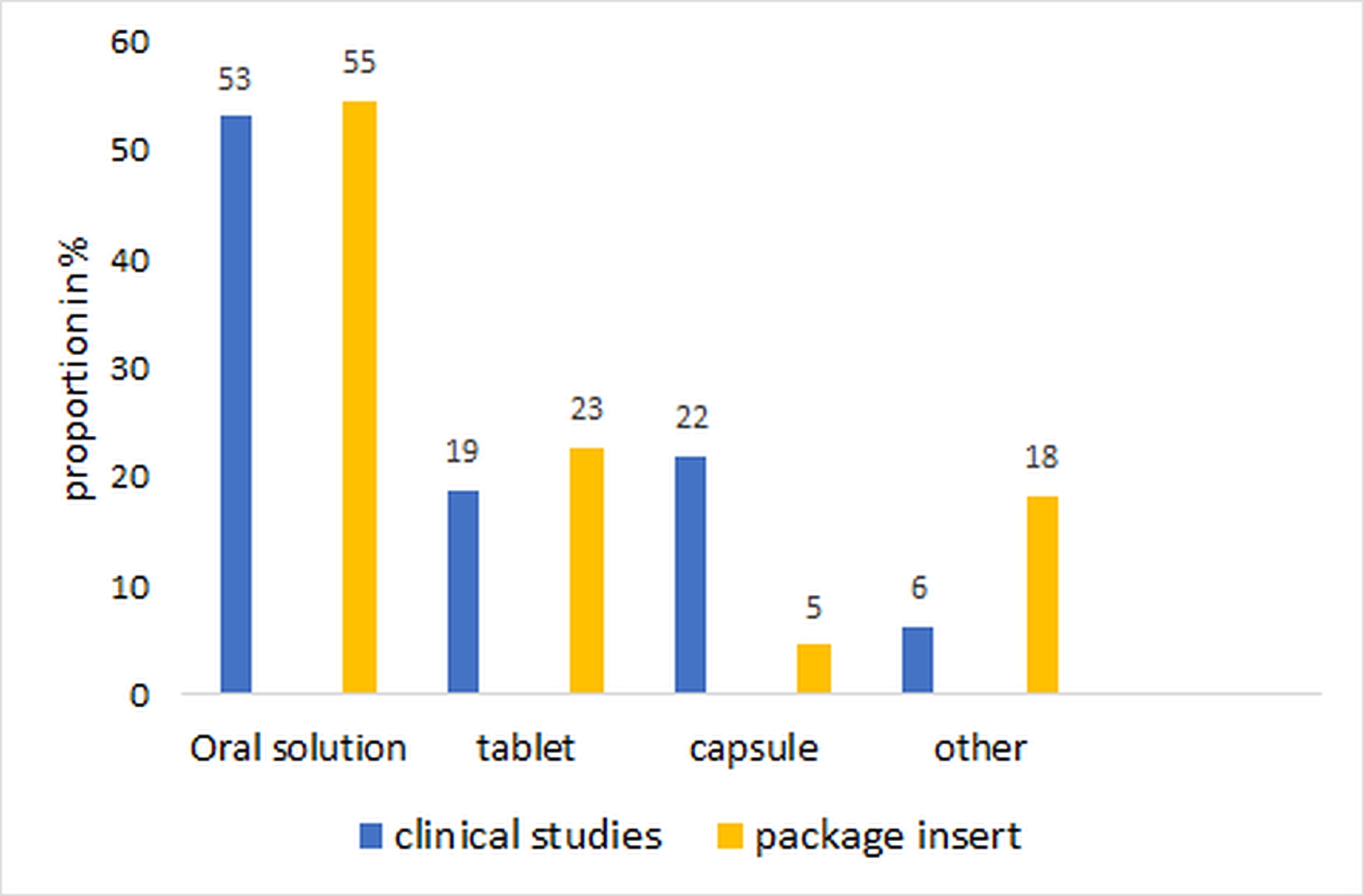


**Fig. S 12:** Comparison of the galenic forms selected in the preparations in clinical studies
 with the preparations available on the market; package insert shown in orange
 and clinical studies shown in blue

**Clinical studies**

**
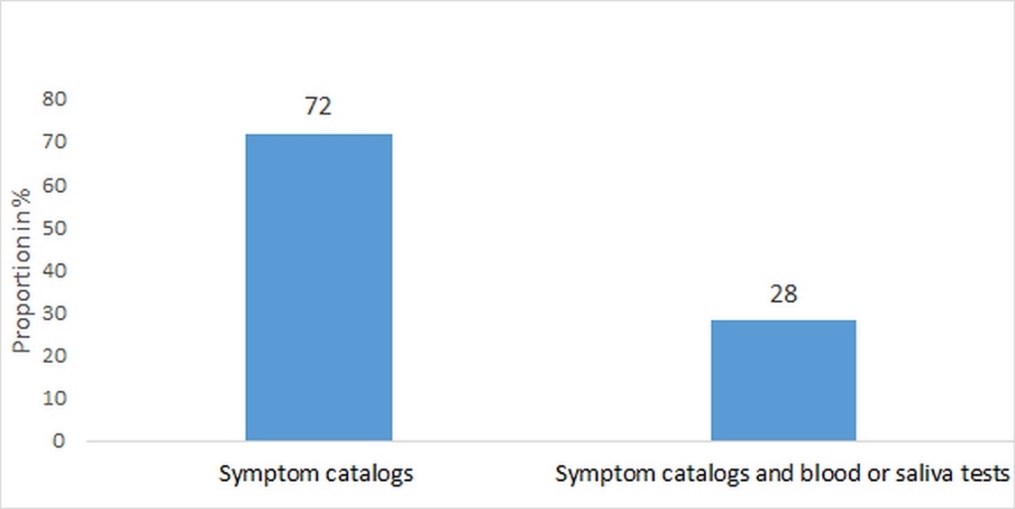
**

**Fig. S 13:** Mode of data collection in the clinical trials plotted as a bar chart in percent

**
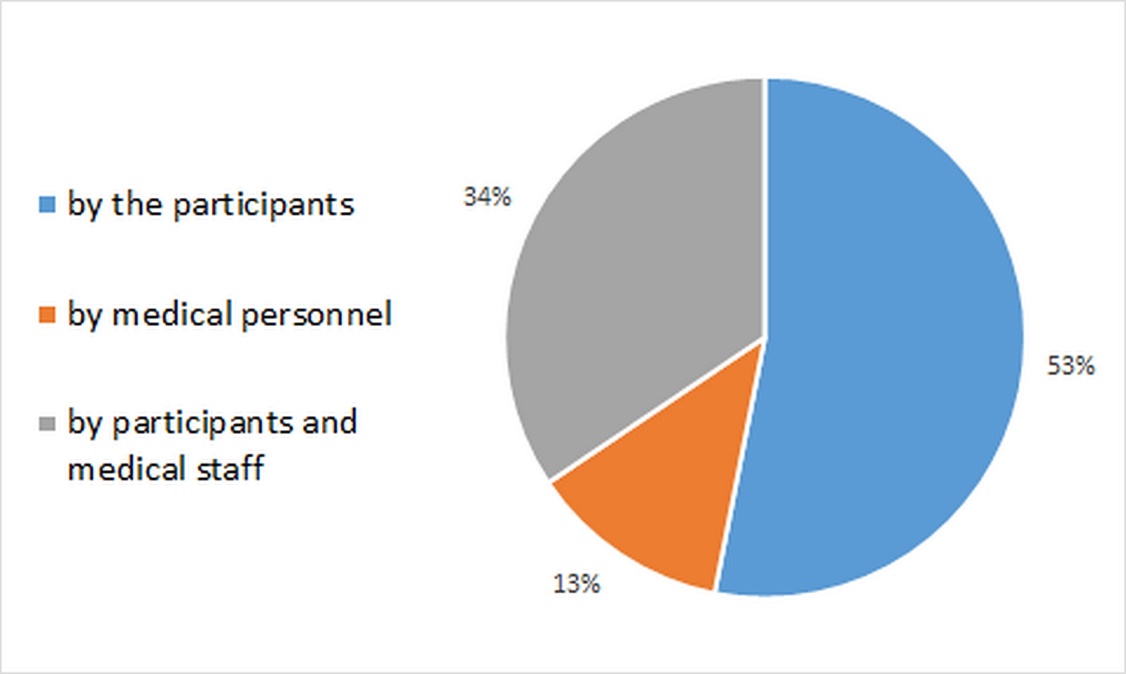
**

**Fig. S 14:** Type of data collection in clinical trials plotted in color as a pie chart
